# Single-strand annealing between inverted DNA repeats: Pathway choice, participating proteins, and genome destabilizing consequences

**DOI:** 10.1371/journal.pgen.1007543

**Published:** 2018-08-09

**Authors:** Sreejith Ramakrishnan, Zachary Kockler, Robert Evans, Brandon D. Downing, Anna Malkova

**Affiliations:** 1 Department of Biology, University of Iowa, Iowa City, IA, United States of America; 2 Indiana University Purdue University Indianapolis, Indianapolis, IN, United States of America; 3 Department of Developmental Biology, Stanford University School of Medicine, Stanford, CA, United States of America; Tufts University, UNITED STATES

## Abstract

Double strand DNA breaks (DSBs) are dangerous events that can result from various causes including environmental assaults or the collapse of DNA replication. While the efficient and precise repair of DSBs is essential for cell survival, faulty repair can lead to genetic instability, making the choice of DSB repair an important step. Here we report that inverted DNA repeats (IRs) placed near a DSB can channel its repair from an accurate pathway that leads to gene conversion to instead a break-induced replication (BIR) pathway that leads to genetic instabilities. The effect of IRs is explained by their ability to form unusual DNA structures when present in ssDNA that is formed by DSB resection. We demonstrate that IRs can form two types of unusual DNA structures, and the choice between these structures depends on the length of the spacer separating IRs. In particular, IRs separated by a long (1-kb) spacer are predominantly involved in inter-molecular single-strand annealing (SSA) leading to the formation of inverted dimers; IRs separated by a short (12-bp) spacer participate in intra-molecular SSA, leading to the formation of fold-back (FB) structures. Both of these structures interfere with an accurate DSB repair by gene conversion and channel DSB repair into BIR, which promotes genomic destabilization. We also report that different protein complexes participate in the processing of FBs containing short (12-bp) versus long (1-kb) ssDNA loops. Specifically, FBs with short loops are processed by the MRX-Sae2 complex, whereas the Rad1-Rad10 complex is responsible for the processing of long loops. Overall, our studies uncover the mechanisms of genomic destabilization resulting from re-routing DSB repair into unusual pathways by IRs. Given the high abundance of IRs in the human genome, our findings may contribute to the understanding of IR-mediated genomic destabilization associated with human disease.

## Author summary

Efficient and accurate repair of double-strand DNA breaks (DSBs), resulting from the exposure of cells to ionizing radiation or various chemicals, is crucial for cell survival. Conversely, faulty DSB repair can generate genomic instability that can lead to birth defects or cancer in humans. Here we demonstrate that inverted DNA repeats (IRs) placed in the vicinity of a DSB, interfere with the accurate repair of DSBs and promote genomic rearrangements and chromosome loss. This results from annealing between inverted repeats, located either in different DNA molecules or in the same molecule. In addition, we describe a new role for the Rad1-Rad10 protein complex in processing fold-back (FB) structures formed by intra-molecular annealing involving IRs separated by long spacers. In contrast, FBs with short spacers are processed by the Mre11-Rad50-Xrs2/-Sae2 complex. Overall, we describe several pathways of DSB promoted interaction between IRs that can lead to genomic instability. Given the large number of IRs in the human genome, our findings are relevant to the mechanisms driving genomic destabilization in humans contributing to the development of cancer and other diseases.

## Introduction

Double strand breaks (DSBs) in DNA result from the interactions of DNA with environmental agents or cellular metabolites, and are a major source of genetic instability (reviewed in [1, 2]). The efficient repair of DSBs acquired over time is critical for cell survival. Since some DSB repair pathways result in mutations and chromosomal rearrangements and others do not (reviewed in [3–5]), the choice of safer repair pathways is important to maintain genomic integrity.

**Fig 1:**
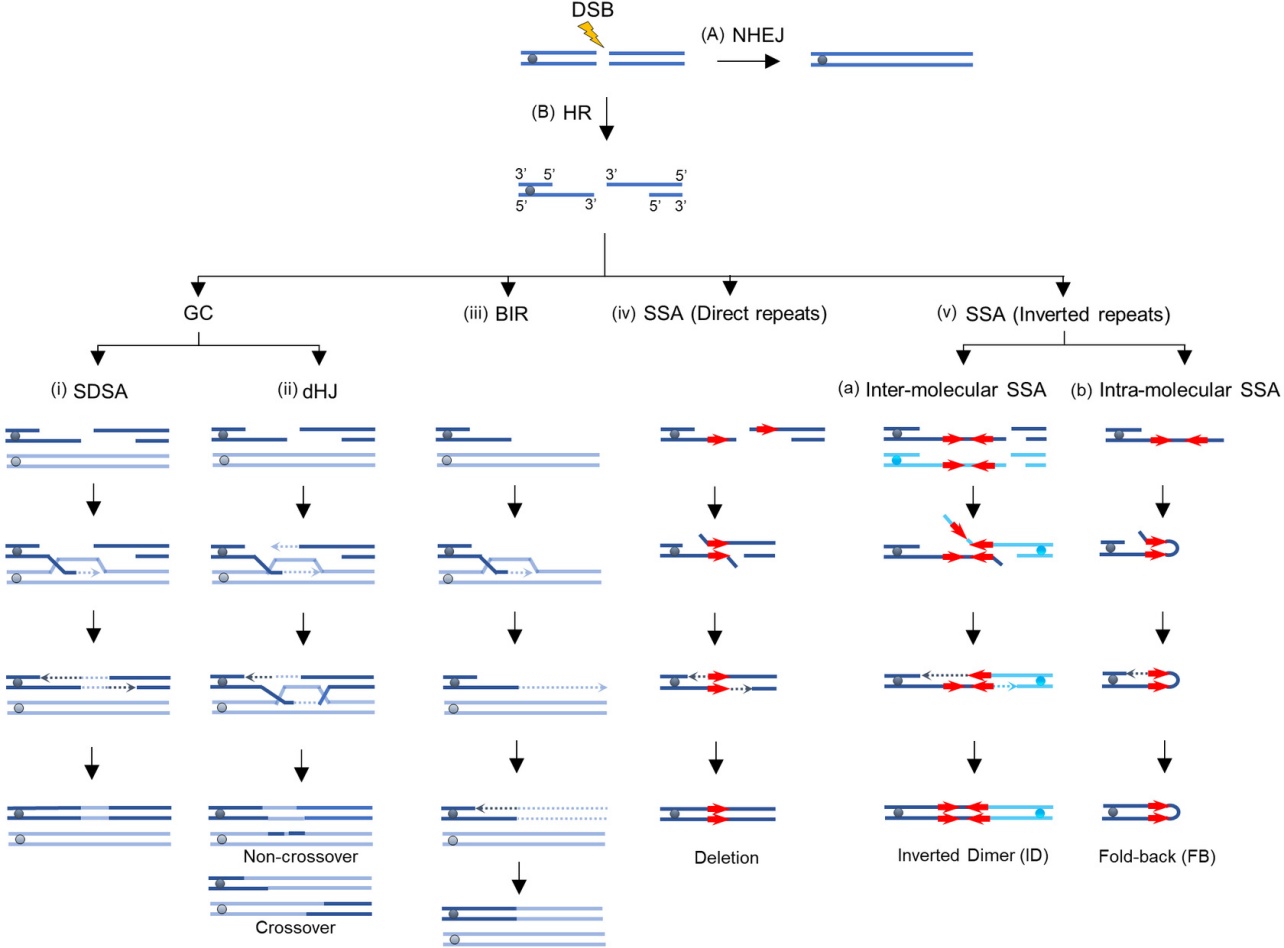
DSB repair pathways. DSBs can be repaired by either **(A)** Non Homologous End Joining (NHEJ) that proceeds by ligation of broken ends or **(B)** Homologous Recombination (HR) where the DSB ends undergo 5’-3’ resection, and repair proceeds via one of the following sub-pathways of HR: (i). SDSA. 3’ end invades and pairs with a homologous donor template producing a displacement loop (D-loop). The 3’ end of the invading strand is used as a primer to initiate new DNA synthesis. The second end of the DSB is resected and pairs with the newly copied strand and initiates a second round of DNA synthesis. (ii) Double Holliday junction (dHJ) pathway; an alternative gene conversion mechanism that synthesizes only a short DNA patch and involves strand invasion step followed by stabilization of a D-loop via “capturing” of the second broken DSB end. Two rounds of synthesis lead to the formation of a dHJ that can be resolved to produce non-crossover or crossover outcomes. (iii) BIR; employed when only one broken end is available for strand invasion, this invasion leads to the initiation of DNA synthesis that proceeds via migrating bubble with asynchronous synthesis of leading and lagging strands and leads to conservative inheritance of newly-synthesized DNA. (iv) SSA between direct DNA repeats. 5’-3’ resection continues until flanking homologous sequences are exposed and annealed to each other. The protruding non-homologous 3’ ends are clipped off and the 3’ ends are used as primers to fill in the gaps. (v) SSA between inverted DNA repeats. (v-a) Inter-molecular SSA between IRs. DSBs occur in two DNA molecules (e.g. sister chromatids), followed by 5’-3’ strand resection, leading to exposure of the IRs as ssDNA, followed by annealing between IRs located on two different DNA molecules. After clipping off the 3’-non-homologous tails, DNA synthesis fills in the gaps leading to the formation of inverted dimers that can also be dicentric. (v-b) Intra-molecular SSA between IRs. 5’-3’ resection exposes the IRs as ssDNA where intra-molecular SSA between IRs occurs. This is followed by clipping off of the non-homologous tails, DNA synthesis and ligation that fills in the gaps ultimately leading to the formation of fold-back (hairpin) structures.

The two main types of DSB repair pathways, non-homologous end-joining (NHEJ) and homologous recombination (HR), are conserved from yeast to humans [2, 6]. NHEJ occurs by re-ligation of broken DNA ends (Fig 1A). HR proceeds via DNA 5’-3’ end resection followed by repair using a homologous DNA sequence (e.g., a sister chromatid or a homologous chromosome) for repair (Fig 1B). HR pathways include synthesis-dependent strand annealing (SDSA), double-Holliday junction (dHJ) repair, break-induced replication (BIR) and single-strand annealing (SSA) [1, 7]. Both SDSA and dHJ repair can lead to gene conversion (GC) (Fig 1B-i,ii). dHJ can also lead to GC associated with crossing-over (CO) and it is frequently used for the repair of meiotic DSBs [2]. While, SDSA predominates in mitotic cells and rarely leads to crossing-over (reviewed in [1, 2]), BIR is a pathway used in situations where only one broken DNA end can find homology for repair (reviewed in [8–10]). BIR is initiated by invasion of a 3’ single-stranded-DNA (ssDNA) end into the homologous sequence, followed by an unusual type of DNA synthesis copying large, often chromosomal sized DNA regions (Fig 1B-iii). Instead of a replication fork, BIR proceeds via a migrating DNA bubble, which causes frequent mutations and large chromosomal changes including deletions, duplications and translocations [10–14]; (reviewed in [8]). Thus, BIR is more deleterious than SDSA, which very rarely leads to genomic instability (reviewed in [1, 2]). SSA proceeds via annealing between DNA repeats that become single-stranded following resection of broken DNA ends. SSA between direct DNA repeats can lead to deletions (Fig 1B-iv), while SSA between inverted repeats promotes the formation of unusual DNA structures namely inverted, sometimes dicentric, chromosome dimers (ID) and fold-backs (FB) (Fig 1B-v) [15–18]. Since the potential for genomic destabilization varies between various DSB repair pathways, it is important to understand how the choice between the pathways is made by living cells. When DSBs are initiated in yeast cells in such a way that both ends possess the homology to the homologous template, repair predominantly proceeds (in more than 90% of the cells) by SDSA or dHJ leading to gene conversion (GC), while repair by BIR is rare [19, 20]. The suppression of BIR leading to the predominance of GC outcomes was proposed to result from a recombination execution checkpoint (REC) that stimulates the choice of healthier DSB repair mechanisms [19]. In addition, several factors, including the type of DNA damage, chromatin structure, the extent of DSB resection, and the age of the cells can influence the choice of DSB repair pathway [21, 22–24], and also reviewed in [1, 7]. For example, older yeast cells use BIR more frequently as compared to younger cells [21]. Also, multiple DNA breaks caused by gamma irradiation of yeast, frequently result in genomic rearrangements via BIR [25]. Such rearrangements could result from breaks introduced at the position of repeated elements, as well as it is also possible that multiple breaks suppress REC, and thus promote the repair of individual breaks via BIR.

The presence of inverted repeats (IRs) in the vicinity of a DSB can also affect DSB repair [17, 26, 27]. IRs are characterized by a symmetry that allows them to switch between inter- and intra-strand base pairing (reviewed in [28]) to form secondary DNA structures, such as cruciforms, hairpins, or fold-backs which can promote genetic instabilities. For example, IRs are implicated in the formation of rearrangements associated with several diseases including cancer, Emanuel syndrome, and X-linked congenital hypertrichosis [29–31]. IRs induce genomic instability by various mechanisms, including impediment of DNA replication at the position of non-B DNA structure formed by IRs or by affecting the repair of DNA. For example, we demonstrated that IRs located *in cis* to HO-induced DSB affected its repair [17]. In particular, we observed that DSBs that were introduced by HO-endonuclease promoted SSA between IRs located 30-kb centromere proximal to these breaks on two different sister chromatids, and this led to the formation of inverted dicentric dimers (Inverted dimers, IDs) [17]. Mitotic breakage of these dicentric chromosomes leads to chromosome rearrangements including translocations, deletions, and duplications. We established that IR-mediated inter-molecular SSA requires Rad52, but is Rad51-independent, similar to SSA involving direct DNA repeats [24]. Our experiments also suggested that inter-molecular SSA can potentially re-route DSB repair from one pathway to another. Specifically, we observed that inter-molecular SSA can successfully compete with DSB repair by allelic BIR and re-route it into ectopic BIR. The problem, however, was that allelic BIR in our previous system was a very slow and inefficient process. A much more interesting question is whether an efficient DSB repair pathway, allelic gene conversion, can also be outcompeted by IR-mediated SSA. However, this question could not be answered using our original experimental system where IRs were located 30-kb away from the DSB-site [17], as it took more than 7 hours to complete SSA due to the need to resect over 30 kb to initiate SSA, whereas GC takes only 2 to 3-hours [20]. In addition, it remained unclear which properties of the IRs played a role in the ability of the IRs to undergo SSA and to promote rearrangements. For example, it has been previously shown that during DNA replication, IRs can form covalently closed hairpin-capped DNA molecules called fold-backs (FBs) [15, 16, 28]. In yeast, the Mre11-Rad50-Xrs2(MRX)/Sae2 complex can cleave FBs with short (12-bp) hairpin loops generating DSBs that can lead to chromosomal rearrangements [16]. The replication of unprocessed FBs leads to the formation of inverted dicentric dimers promoting breakage-fusion-bridge cycles [15, 16] leading to chromosomal rearrangements including DNA amplifications, deletions, and translocations [15, 18]. While the formation of FBs by IRs during replication is well-studied [18], the formation and processing of FBs following DSB induction is not well investigated. It remains unknown whether formation of FBs depends on the structure of IRs and how FBs containing long hairpin loops are formed and processed. Also, the molecular mechanisms of IR-mediated genome destabilization remain unclear.

We here present a new experimental system, with IRs located close (~3-kb) to the DSB site. Using this system, we tested whether DSBs can promote IR-mediated SSA, and whether it can compete with pathways leading to the formation of GC. Our data suggests that IRs can participate in both inter- and intra- molecular SSA leading to the formation of inverted dicentric chromosome (ID) and fold-back (FB) structures, respectively. We show that the choice between inter- and intra- molecular SSA is determined by the length of the spacer separating the IRs. In strains where IRs are separated by a long 1-kb spacer, inter-molecular SSA is more efficient, while intra-molecular SSA dominates in strains containing IRs separated by a short 12-bp spacer. We also demonstrate that different protein complexes are involved in the processing of FBs containing long and short hairpin loops. Specifically, FBs with short (12-bp) hairpin loops are processed by the MRX-Sae2 complex, whereas FBs with long (1-kb) loops are processed by the Rad1-Rad10 complex. In addition, we demonstrate that the presence of IRs in the vicinity of DSBs promotes the repair of these DSBs via BIR, while decreasing the frequency of GC. Taken together, our results indicate how inverted DNA repeats influence the choice and outcome of DSB repair and can result in chromosomal rearrangements.

## Results

### Inverted repeats channel repair of a nearby DSB into SSA pathway between sister chromatids

**Fig 2:**
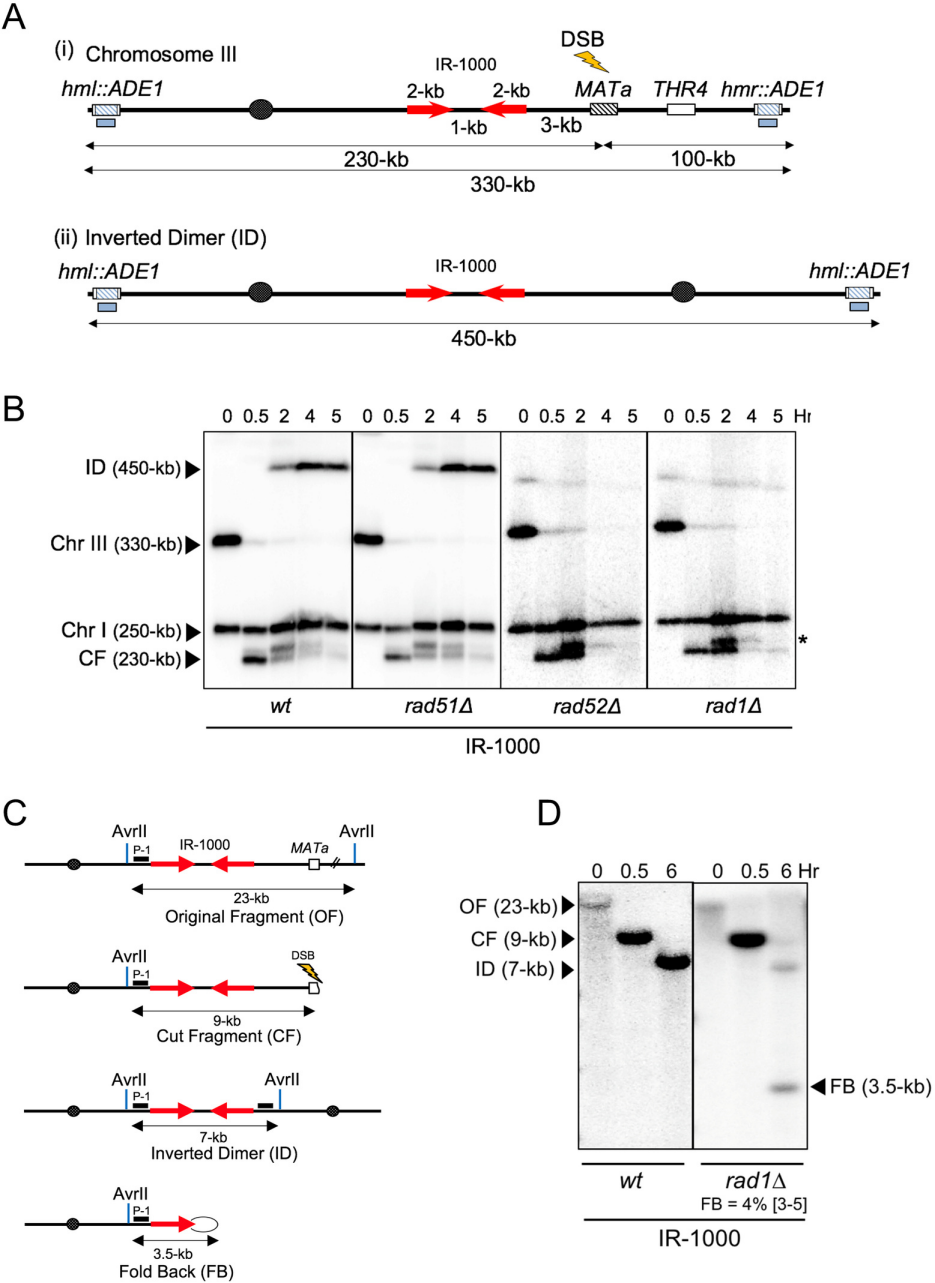
SSA between IRs separated by 1-kb spacer. **(A)** Experimental system to study SSA between IRs. (i) Two 2-kb long IRs separated by 1-kb long spacer (IR-1000) were inserted into Chromosome III (Chr III), centromere proximal to *MAT***a** where DSB was induced by galactose-inducible HO endonuclease. (ii) The structure of Inverted dicentric dimer (ID) resulting from SSA between IRs located on the two different sister chromatids, as demonstrated in Fig 1v-a. Blue boxes under the chromosomes indicate the locations of the *ADE1*-specific probe on Chr III. **(B)** DSB repair analysis using CHEF gel electrophoresis of DNA isolated from wt (*RAD1*), *rad51Δ*, *rad52Δ*, *and rad1Δ* strains before (0 hour (Hr)), as well as 0.5, 2, 4, and 5 hours following DSB induction. The full-length Chr III, ID, cut fragment (CF) of Chr III generated by HO-induced DSB, and intact Chr I were detected by hybridization with *ADE1*-specific probe, and DNA fragment sizes are indicated in parenthesis. Asterisks indicate the shifted cut-fragment bands following DSB resection as described in [17, 86]. **(C)** The structure of original AvrII restriction fragments (OF) of chromosome III in IR-1000 strain (2-kb-long IRs separated by 1-kb-long spacer) and its derivatives following DSB: CF, ID, and FB. The location of hybridization probe P-1, specific to *RBK1* sequence is indicated by black box. **(D)** Southern blot analysis of DSB repair in IR-1000 wt strain and its *rad1Δ* derivative following hybridization to probe P-1. The positions and corresponding sizes of OF, CF, ID and FB following AvrII restriction digestion of DNA isolated before (0-hr), as well as 0.5 and 6 hours following DSB induction are indicated. The median efficiency of FB formation (%) and the range of the median [in brackets] are indicated (see S1 Fig and Materials and Methods for details). Every gel was run with appropriate molecular-size markers that allowed to estimate the sizes of every band shown in each figure.

Our goal was to examine the effect of inverted DNA repeats on the repair of nearby DSBs. To accomplish this, we generated a haploid strain (IR-1000) with an IR consisting of two 2-kb-long sequences separated by a 1-kb-long spacer and located 3 kb centromere proximal to *MAT***a**, on chromosome III (Fig 2A-i). This IR was shorter than the Ty elements that we previously used to describe IR-mediated SSA [17], but was within a length range that was previously used by various groups to study SSA genetics and IR-mediated GCRs [18, 25, 32–34]. The IR in this study was created by inserting a 2-kb long copy of *PHO87* gene in an inverted orientation next to the endogenous *PHO87* gene originally located at this chromosomal position.

**Fig 3:**
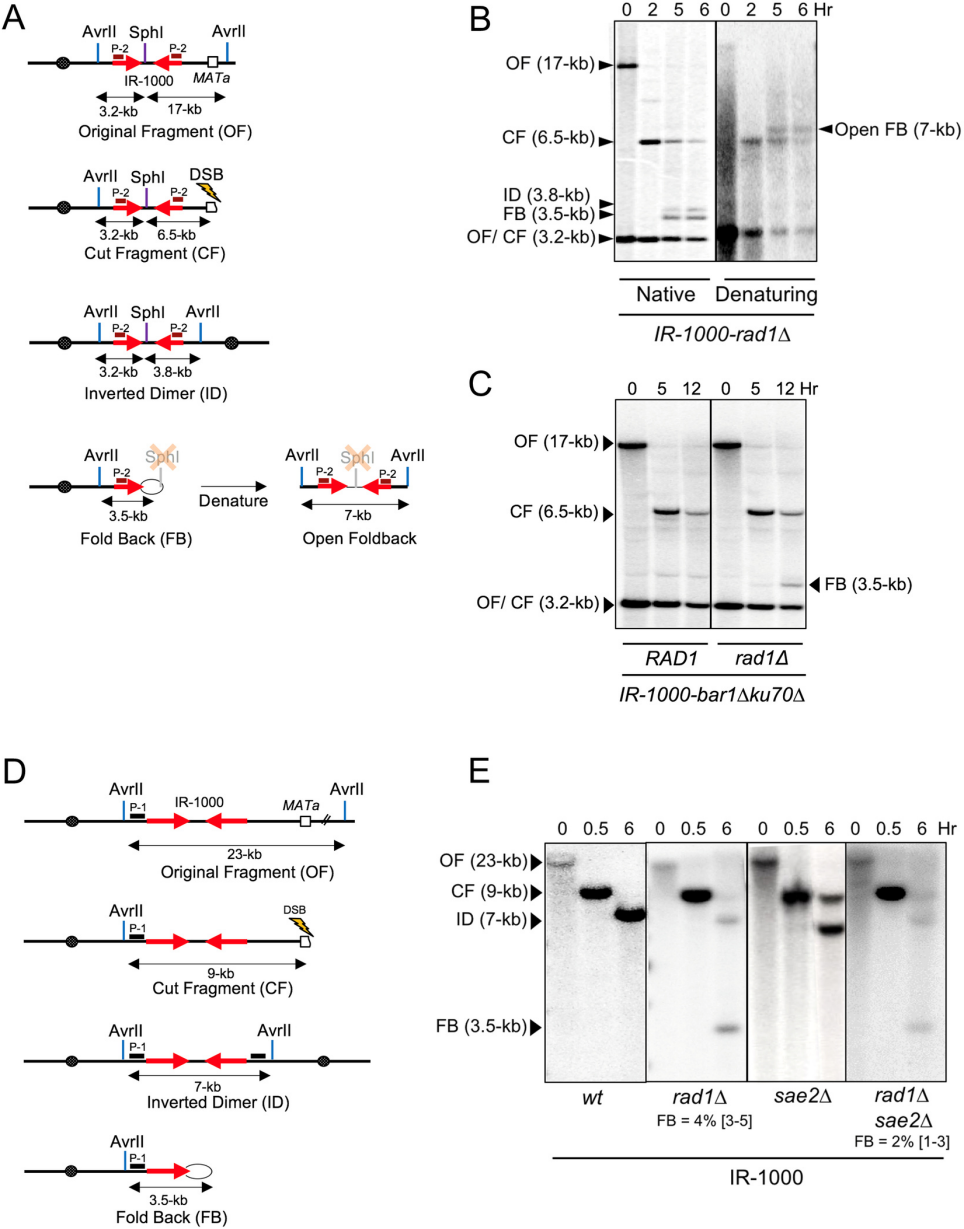
The role of Rad1 in processing of fold-backs with 1-kb spacers. **(A)** The schematics of AvrII /SphI digest of chromosome III in IR-1000 (OF) and its derivatives, including: CF, ID, FB, and “open” fold-back resulting from denaturation of FB. The location of probe P-2, specific to *PHO87* sequence, is indicated by brown box. **(B)** Southern blot analysis of DSB repair in *rad1Δ* derivative of IR-1000 strain following native or denaturing gel electrophoresis after AvrII /SphI digest of DNA isolated before (0 hr), or 2, 5, 6 hours after DSB induction, followed by hybridization with probe P-2. **(C)** FB formation in IR-1000 arrested at G1 stage of the cell cycle. DNA extracted from *RAD1* or *rad1Δ* at 0-hr (prior to DSB induction) and 5 or 12-hr post-DSB-induction, digested with AvrII and SphI followed by native gel electrophoresis and hybridization with probe P-2. **(D)** Schematics of AvrII digested ChrIII and its derivatives; OF, CF, ID and FB in IR-1000. **(E)** Analysis of FB and ID formation in IR-1000 (wt) and its derivatives by AvrII digest, native gel electrophoresis, and hybridization with probe P-1. The median efficiencies of FB formation (%) and the range of the median [in brackets] are indicated (see S1 Fig and Materials and Methods for details).

**Fig 4:**
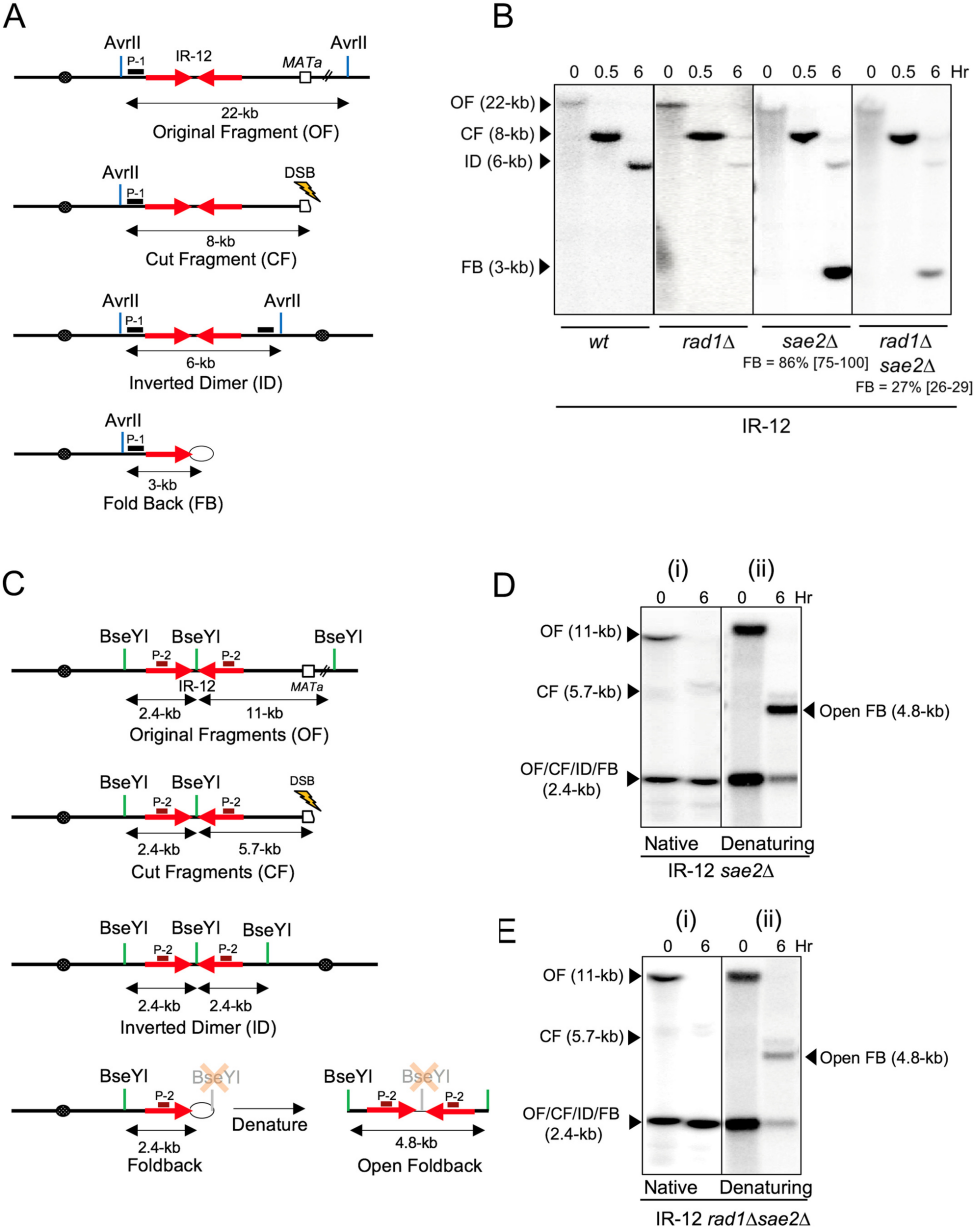
Inverted dimers and fold-backs in IR-12. **(A)** Schematics of AvrII- digested Chr III in IR-12 (OF) and its derivatives: CF, ID, FB (location of probe P-1 is indicated by black box). **(B)** DSB repair in IR-12 (wt, *rad1Δ*, *sae2Δ* and *rad1Δsae2Δ)* following AvrII digest and hybridization to probe P-1. The respective positions of, CF, ID and FB are indicated. The median efficiencies of FB formation (%) and the range of the median [in brackets] are indicated (see S1 Fig and Materials and Methods for details). **(C)** The schematics of BseYI restriction fragments of Chr III in original IR-12 and its derivatives following DSB: OF, CF, ID, FB and Open-FB (formed by denaturation of FB). The location of hybridization probe P-2 is indicated by brown box. **(D)** Southern blot analysis of DSB repair in *sae2Δ* derivative of IR-12 using native and denaturing gel electrophoresis (Schematics in Fig 4C) and hybridization to probe P-2. The positions and corresponding sizes of OF, CF, ID, FB and Open-FB following BseYI digestion of DNA isolated before (0-hr) and 6-hr following DSB induction are indicated. **(E)** Southern blot analysis of DSB repair in *rad1Δsae2Δ* derivative of IR-12 using native and denaturing gel electrophoresis (schematics in Fig 4C) following hybridization to probe P-2.

**Fig 5:**
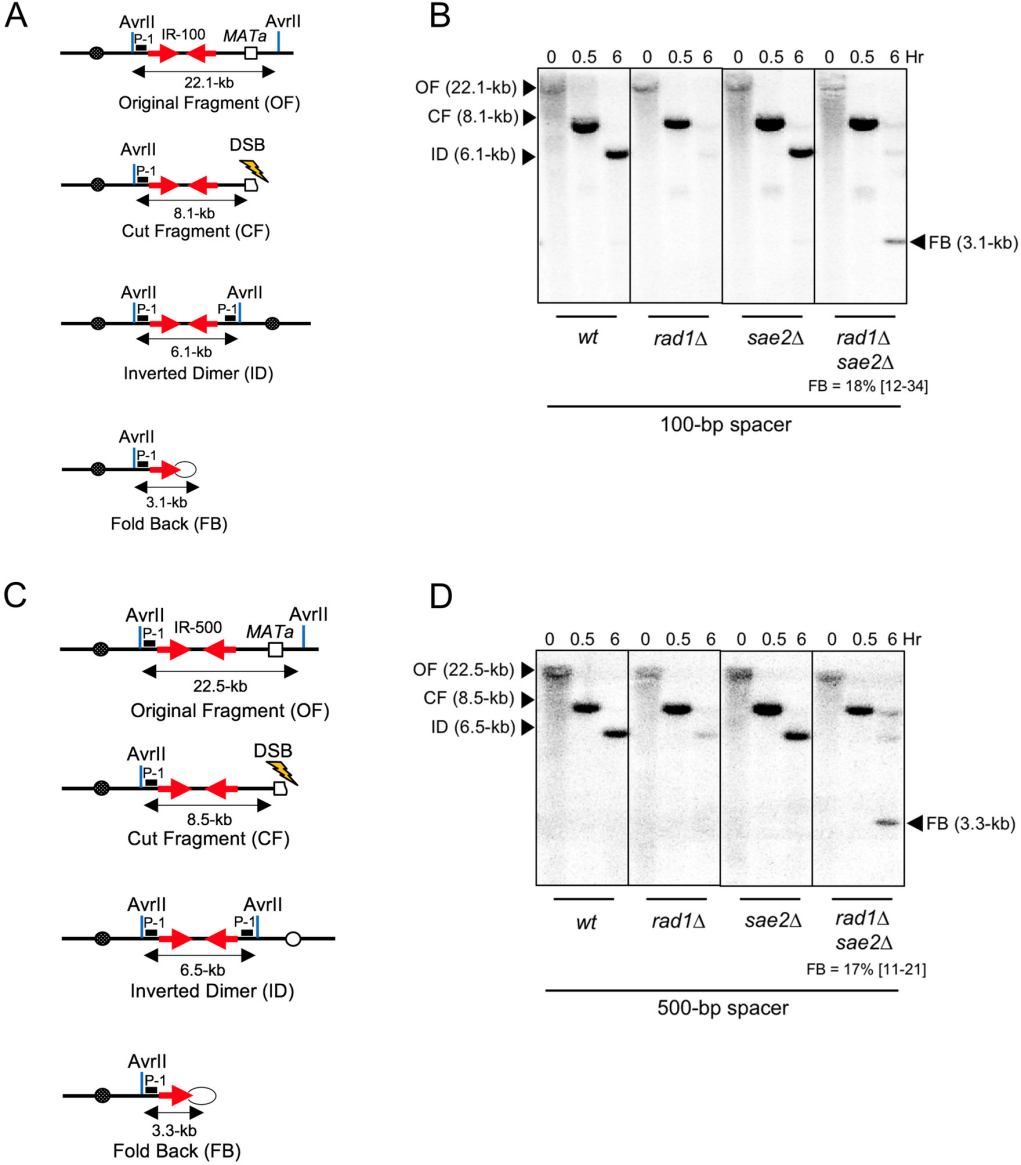
Formation of ID and FB structures in strains with 100-bp and 500-bp spacer between IRs. **(A)** The structures of AvrII restriction fragments of Chr III (OF) in IR-100 strain (2-kb IR separated by 100-bp spacer) and its derivatives following DSB: CF, ID, FB. **(B)** Southern blot analysis of DSB repair in wt, *rad1Δ*, *sae2Δ* and *rad1Δsae2Δ* derivatives of IR-100 following hybridization to probe P-1 (depicted by black box in Fig 5A) **(C)** The schematics of AvrII digest of Chr III (OF) in IR-500 strain (2-kb IR separated by 500-bp spacer) and its derivatives following DSB: CF, ID, FB. **(D)** Southern blot analysis of DSB repair in wt, *rad1Δ*, *sae2Δ* and *rad1Δsae2Δ* derivatives of IR-500 strain following hybridization to probe P-1. The median efficiencies of FB formation (%) and the range of the median [in brackets] are indicated (see S1 Fig and Materials and Methods for details).

A DSB was introduced at *MAT***a** by the HO-endonuclease expressed under the control of a galactose-inducible promoter [35]. To follow the kinetics of DSB repair, samples from yeast liquid culture were collected at various time-points after DSB induction, and repair was analyzed using contour-clamped homogeneous electric field (CHEF) gel electrophoresis followed by Southern blot hybridization using *ADE1*-specific sequence as a probe (Fig 2A-i, ii, Fig 2B). A 450-kb DSB repair product was accumulated between 2 and 5 hours following DSB induction (Fig 2B, wt). Based on the size of this repair product, we hypothesized that it represents an inverted dimer (ID) formed by single strand annealing (SSA) between IRs located on different sister chromatids, similarly to previously described [17] (Fig 2A-ii, see also Fig 1B-v-a). This was confirmed by digesting genomic DNA with AvrII restriction enzyme, followed by gel electrophoresis and Southern blot hybridization using a probe specific to *RBK1* gene, a sequence located near and centromere proximal to the IRs (Fig 2C, probe P-1, 2D). We observed that the 23-kb original fragment (OF), present in chromosome III before the induced DSB in the 0-hr time-point, disappeared after the induction of a DSB (Fig 2C–OF, 2D-wt), whereas at the 0.5-hr time-point a 9-kb fragment corresponding to the DSB cut fragments (CF) appeared, and finally at the 6-hr time point a 7-kb fragment appeared consistent with the molecular-size of ID (SSA repair product) fragment. The amount of ID product detected at 6 hours after DSB induction was 76% of the amount of broken chromosome-III (cut-fragment, CF) in wt cells (Fig 2D-wt, S1A Fig), indicative of a high efficiency of this ID pathway resulting from inter-molecular SSA. The cut-fragment band (0.5-hr time-point post DSB induction) was used to calculate the amounts of repair products because it represented the total amount of the chromosome that was broken, following DSB induction. In addition, the high molecular weight of the original-chromosome fragment (OF) at 0-hr time-point made the Southern-transfer of this fragment inefficient and therefore unreliable for calculations. Overall, our results indicated that inter-molecular SSA leading to the formation of ID is the predominant pathway of DSB repair in this haploid strain. Similar results were obtained in the same strain in the presence of nocodazole, which prevents mitotic divisions and excludes the possibility of dicentric formation after replication of a fold-back structure resulting from intra-molecular annealing between inverted repeats. As predicted, formation of IDs occurred much faster in the strain containing IRs that are located 3 kb away from *MAT***a** compared to what we observed in our previous experimental system where IRs were located > 30-kb away from the DSB site [17]. The formation of IDs requires Rad52, but not Rad51 (Fig 2B), consistent with the current models for SSA [24, 36]. We also asked whether ID formation requires the flap endonuclease Rad1 known to be involved in SSA between direct DNA repeats [37]. In the absence of Rad1, the level of IDs was reduced more than 10-fold as compared to *RAD1* (wt) strain (Fig 2B-5-hr wt vs *rad1Δ*). The analysis of genomic DNA digested with AvrII endonuclease (Fig 2C) also showed that the amount of ID also was significantly decreased in *rad1Δ* (3%) compared to *RAD1* (wt, 76%) (Fig 2D; see also S1A Fig). Overall, we conclude that inter-molecular SSA between IRs is a predominant pathway of DSB repair when the DSB occurs near IRs. Surprisingly, the analysis of DSB repair in *rad1Δ* revealed the formation of an additional 3.5-kb band at 6-hr time-point (Fig 2D-*rad1Δ*). We hypothesized that this fragment represented a fold-back hairpin molecule formed by annealing of IRs within the same resected 3’-single stranded DNA with the spacer between IRs forming a single stranded loop at the tip of the hairpin (Fig 1B-v-b), similar to those reported previously when inverted repeats were included in a single-stranded DNA region [15, 16] although the role of Rad1 in the accumulation of FBs was not reported before.

### The role of Rad1 in the processing of fold-back structures containing long spacers

To confirm that the 3.5-kb repair product detected in IR-1000-*rad1Δ* strain (Fig 2D) had a structure expected of a FB molecule (Fig 1B-v-b), we used a combination of native and denaturing gel electrophoresis of genomic DNA digested with AvrII and SphI enzymes (Fig 3A and 3B). The two AvrII sites flanked the IR region, while the SphI site was located in the spacer DNA between the IRs and was expected to be refractory to cutting by Sph1 following FB formation due to single-stranded nature of the loop formed by the spacer DNA between IRs in the FB molecule (Fig 1B-v-b, Fig 3A-FB). Due to these reasons, such an FB-fragment is expected to open into an open-FB molecule with a size that is twice the size under denaturing conditions compared to the size of the FB molecule under native electrophoresis conditions. Indeed, denaturing gel electrophoresis shows a new 7-kb band, expected from the denaturing of a 3.5 kb hairpin molecule (Fig 3A and 3B-denaturing gel). Moreover, the 3.5-kb band was consistently detected in our experiments in IR-1000-*rad1Δ* strain at 6-hr time-point following hybridization to either the *RBK1*-specific probe P-1 (Fig 2C and 2D-*rad1Δ)* or the *PHO87*- specific probe P-2 (Fig 3A and 3B-native gel). Further, the 3.5-kb band did not show hybridization to a probe specific to the 3’-flap sequence, including the region located between IRs and *MAT***a** (S1B Fig, probe P-3), which was expected to be removed prior to FB formation (S1C Fig). These results suggest that the 3.5-kb repair product observed in IR-1000-*rad1Δ* strain has a structure that is consistent with FB hairpin molecule. Based on our data we propose that Rad1 is responsible for the processing of FBs with 1-kb loops, and when Rad1 is missing, FBs accumulate in the cells.

Another possibility was that the accumulation of FBs in the absence of Rad1 in our IR-1000-*rad1Δ* strain resulted from the channeling of inter-molecular SSA intermediates that are incapable of forming IDs in the absence of Rad1 into intra-molecular SSA generating FBs. This possibility was addressed by repeating the experiment in cells arrested at the G1 stage of the cell cycle when inter-sister SSA cannot occur. We asked whether FBs can be detected in RAD1^+^ (wt) cells that are arrested in G1 stage where there is no sister chromatid to form IDs. The experiment was performed in *RAD1*^*+*^ and *rad1Δ* strains with IRs separated by a 1-kb spacer that were also *bar1Δ* (to facilitate cell cycle arrest at G1 by α-factor pheromone) [38]. The complication for our experiment was that resection of DSB ends required for the formation of FBs is normally defective in cells arrested at G1. To overcome this problem, we made the cells *ku70Δ* similar to described in ([39]), and this restored resection to an extent. Following the experiment in G1-arrested cells, we detected accumulation of FBs in *rad1Δ*, but not in *RAD1* cells (Fig 3A and 3C). It took about 12-hours following DSB induction to detect FBs in this *rad1Δ* strain. This slower rate of FB formation likely resulted from slower DSB resection in G1-arrested cells. The key result is that even in G1-stage of the cell cycle where no inter-molecular SSA could occur, we still detected an accumulation of FBs in *rad1Δ*, but not in *RAD1* cells. These results suggested that FB represents an independent pathway of its own rather than the result of channeling from an unsuccessful ID formation and supported our hypothesis of direct involvement of Rad1 in processing of FB structures containing long spacers.

To confirm that processing of FBs by Rad1 is not sequence-specific, we modified the construct by replacing its inter-IR spacer with DNA of bacteriophage lambda that was 1-kb or 1.5-kb long (S2 Fig). In both these strains, FBs were detected in the *rad1Δ* background, but not in the isogenic *RAD1*^*+*^ strains. This is consistent with Rad1 being involved in the processing of FBs with long (≥1-kb) ssDNA loops regardless of their DNA sequence.

### Different protein complexes process FBs containing long and short ssDNA loops

The novel role of Rad1 in processing of FBs that we observe in our IR-1000-*rad1Δ* strain is similar to the known function of the MRX-Sae2 complex in FB processing [16]. However, we did not observe FBs in the *sae2Δ* derivative of our IR-1000 strain (Fig 3D and 3E- *sae2Δ*). This could be due to the difference in the properties of IRs in our strain as compared to IRs used in previously published strains [16]. In particular, the role of MRX-Sae2 in FB processing was previously described in strains where IRs were separated by short (~12-bp) spacers, while the distance between IRs in our strain is 1000 bp. We therefore reduced the length of the spacer between IRs in our strain from 1000 bp to 12 bp generating the IR-12 strain. Consequently, we observed a robust accumulation of FBs in *sae2Δ* derivative, but not in *rad1Δ* derivative of IR-12 strain at 6-hours following DSB induction (Fig 4A and 4B- *sae2Δ*, *4B-rad1Δ*). In particular, at 6-hr after DSB induction, the intensity of a 3-kb FB band observed in IR-12-*sae2Δ* was 86% of the intensity of the cut fragment measured at 0.5-hr (Fig 4B-*sae2Δ*, S1D Fig), demonstrating that the FB is the predominant outcome for IR-12. The FB structure of this product was also confirmed by a combination of native and denaturing gel electrophoresis of genomic DNA digested with BseYI restriction enzyme (Fig 4C and 4D). In particular, the FB molecule formed a 2.4-kb band following the native gel electrophoresis, but on denaturing gel electrophoresis a 4.8 kb band was observed. This is expected from the denaturing and opening of a 2.4-kb hairpin shaped FB molecule to form 4.8-kb open-FB molecule (Fig 4C). (Please note that some 2.4-kb band remained present in 6-hr sample following denaturing gel electrophoresis as the remaining OF, CF & ID also generate 2.4-kb band).

We also observed the accumulation of FB structures in IR-12-*rad1Δsae2Δ* strain (Fig 4B). However, the amount of FBs accumulated in IR-12-*rad1Δsae2Δ* was 3-fold lower as compared to the amount of FBs accumulated in IR-12-*RAD1sae2Δ* (Fig 4B, S1D Fig). The fold-back structure of the repair products accumulated in *rad1Δsae2Δ* was also confirmed by a combination of native and denaturing gel electrophoresis (Fig 4C and 4E) as described before. This demonstrates that Rad1 is also involved in the removal of 3’-flaps prior to the formation of a covalently-closed hairpin FB structure.

### Redundant roles of Rad1/Rad10 and MRX-Sae2 in processing of FBs with intermediate-sized spacers

Since *rad1Δ* and *sae2Δ* showed opposite effects on accumulation of FBs for IRs with large and small spacers, we examined their effect on IRs with intermediate spacers. In particular, we analyzed DSB repair in strains containing IRs separated by 100-bp and 500-bp spacers. In these experiments, neither *sae2Δ* nor *rad1Δ* single mutants showed accumulation of FBs (Fig 5A–5D). Accumulation of FBs was detected only in double *rad1Δsae2Δ* mutants. The yield of FB product in *rad1Δsae2Δ* strains containing 100-bp and 500-bp spacers was respectively 18% and 17% of the broken chromosome III (S1D Fig). Thus, our data suggests that although Rad1 and MRX-Sae2 have unique roles in processing FBs with long (~1-kb) and small (~12-bp) loops respectively, both proteins could process FBs with intermediate size loops.

### The length of the spacer separating IRs affects the choice of DSB repair pathway

In our experiments, the length of the spacer separating IRs determined not only the proteins involved in FB processing, but also the choice between inter-molecular SSA (leading to ID formation) and intra-molecular SSA (leading to FB formation). In particular, the change of the spacer length from 1-kb to 12-bp led to the decrease in the amount of IDs from 76% to 28% (Figs 3E-wt, 4B-wt; S1A Fig-IR1000 & IR-12), and to a corresponding increase in FB formation from 4% in 1kb-*rad1Δ* to 86% in 12bp-*sae2Δ* (Figs 3E-*rad1Δ*, 4B-*sae2Δ*; S1D Fig-IR-1000-*rad1Δ* & IR12*-sae2Δ*). The intermediate distance between IRs (100-bp or 500-bp), was associated with intermediate level of IDs (~50% of the broken chromosome III (Fig 5B-wt, 5D-wt, S1A Fig)), and intermediate amounts of FBs (~18% of broken chromosome III (S1D Fig, Fig 5B-*rad1Δsae2Δ*, Fig 5D-*rad1Δsae2Δ*)). Together, these results suggest that ID is the prominent repair product in strains with a long (1-kb) spacer, while FB is predominant in strains with small (12-bp) spacer. This difference in the efficiency of FB formation at least in part explains why the effect of *sae2Δ* on FB formation, observed in strains with small (12-bp) spacer, was so much more pronounced as compared to the effect of *rad1Δ* that we observed in strains with long (1- kb) spacer.

### Proteins involved in the formation and processing of FBs

**Fig 6:**
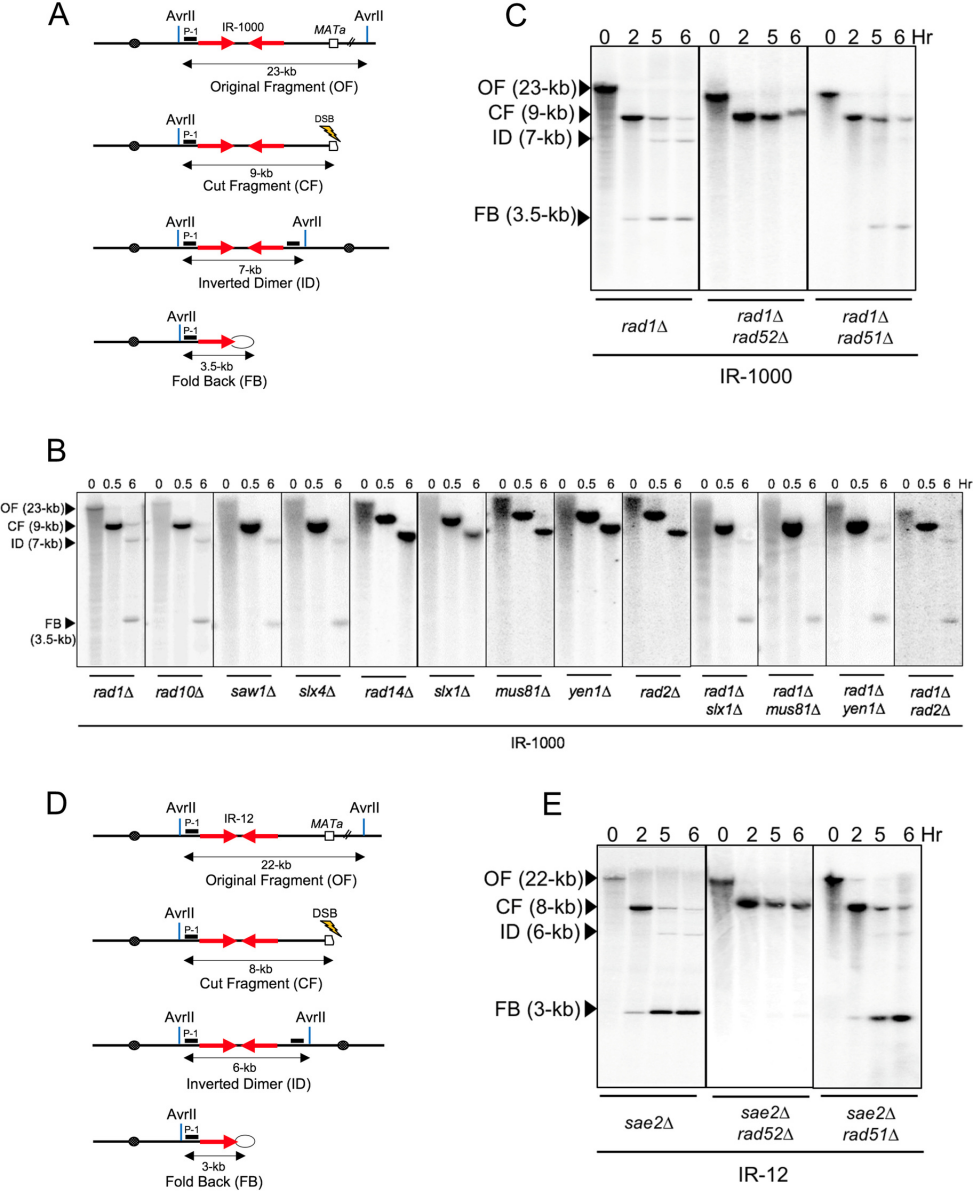
Genetic control of FB formation. **(A)** Schematics of AvrII digest of Chr III (OF) in IR-1000 and its DSB-induced derivatives including: CF, ID, FB. **(B)** Analysis of ID and FB formation in IR-1000 containing one of the following deletions: *rad1Δ*, *rad10Δ*, *saw1Δ*, *slx4Δ*, *rad14Δ*, *slx1Δ*, *mus81Δ*, *yen1Δ*, *rad2Δ*, *rad1Δslx1Δ*, *rad1Δmus81Δ*, *rad1Δyen1Δ or rad1Δrad2Δ* using AvrII digest of DNA, gel electrophoresis and hybridization with probe P-1 (indicated in Fig 6A by black box). **(C)** The role of *RAD52* and *RAD51* in FB formation in IR-1000 derivatives analyzed by AvrII digest followed by hybridization with probe P-1. **(D)** The schematics of AvrII digest of Chr III in IR-12 strain (OF) and its DSB-induced derivatives: CF, ID, FB. **(E).** Analysis of ID and FB formation in *sae2Δ*, *sae2Δrad52Δ*, and *sae2Δrad51Δ* derivatives of IR-12 following AvrII digestion and hybridization with probe P-1.

Several proteins, including Rad10, Saw1 and Slx4 are known to collaborate with Rad1 in the removal of 3’-flaps during SSA involving direct repeats [40, 41]. We used the IR-1000 strain with deletion of *RAD10*, *SAW1*, or *SLX4* genes to ask whether the corresponding genes also accumulate FBs similar to that observed in *rad1Δ* strain, suggesting these proteins assist Rad1 for processing FB structures. We observed that each of these deletions led to the accumulation of unprocessed FBs (Fig 6A and 6B). On the other hand, deleting *RAD14*, which is involved (along with *RAD1*) in nucleotide excision repair (NER) (reviewed in [42]) did not produce detectable FBs (Fig 6B). Thus, processing of FBs in our experiments requires a 3’-flap removal by Rad1-Rad10 complex rather than its role in NER. Additionally, since the amount of FBs accumulated in *rad1Δ* in our experiments was low (4% (S1D Fig)), we asked whether other known yeast nucleases may work in parallel with Rad1-Rad10 in processing FBs. We expected that deleting the gene encoding for such a nuclease may also lead to accumulation of FBs, while deletion of this gene in a *rad1Δ* background will increase the amount of FBs as compared to the *rad1Δ* alone. We observed that deleting genes encoding several known yeast nucleases (*SLX1*, *MUS81*, *YEN1* and *RAD2* (reviewed in [43, 44]) did not lead to FB accumulation, and did not visibly change the amount of FB accumulated when these genes were deleted in *rad1Δ* background (Fig 6B). Therefore, it is unlikely that Slx1, Mus81, Yen1 or Rad2 participate in the processing of FB structures. This result also suggested that these four endonucleases were unlikely to be the unknown proteins involved in the removal of 3’- flaps in the absence of Rad1.

Finally, we asked whether Rad52, known to promote annealing between single-stranded homologous DNA in yeast [45] is required for the formation of fold-back structures. We observed that deleting *RAD52* prevented the accumulation of FBs in both IR-12-*sae2Δ* and IR-1000-*rad1Δ* strains, where accumulation of FBs occurred otherwise in IR-12-*sae2Δ* and IR-1000-*rad1Δ* strains containing *RAD52* (Fig 6A, 6C, 6D and 6E). Importantly, formation of FBs was not affected by deleting *RAD51*, encoding a strand invasion protein, in IR-12-*sae2Δ* and IR-1000-*rad1Δ* strains (Fig 6C and 6E). We conclude that Rad52 (but not Rad51) is required for DSB-induced annealing between inverted repeats located in the same DNA molecule regardless of the distance separating the repeats.

### IRs shift DSB repair from GC to BIR

We have previously demonstrated that DSBs introduced in such a way that both broken ends bear homology to the allelic position of the homologous chromosome are predominantly repaired by pathways leading to allelic GC, and rarely by BIR, a deleterious DSB repair mechanism that operates when only one of the broken DNA ends is available for repair (reviewed in [8, 9, 46, 47]). Here we asked whether the presence of IRs close to a DSB position leads to IR-mediated SSA and promotes BIR.

**Fig 7:**
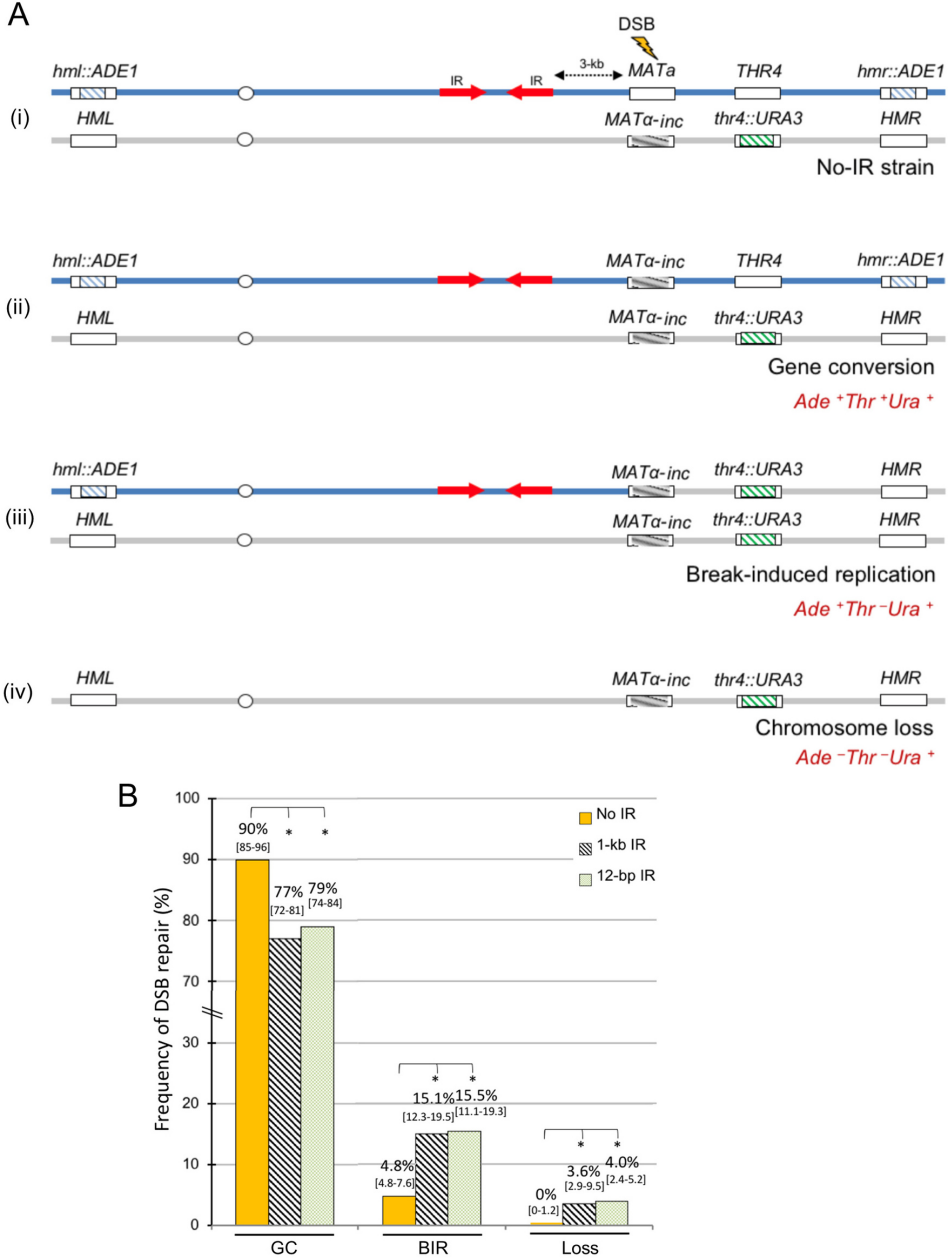
IRs channel DSB repair into BIR and chromosome loss. **(A) (i)** Schematics of diploid experimental strain where a DSB is induced by a galactose-inducible HO-endonuclease at *MAT***a** locus on the chromosome III (top blue). The *MATα-inc* chromosome (bottom grey) is resistant to cutting by HO. The *HML* and *HMR* in the *MAT***a** containing chromosome are replaced by *ADE1*. **(ii)** Gene conversion outcome—Ade^+^ Thr^+^ Ura^+^. **(iii)** Break induced replication outcome: Ade^+^ Thr^-^ Ura^+^. **(iv)** Chromosome loss outcome: Ade^-^ Thr^-^ Ura^+^. **(B)** The frequency of gene conversion (including gene conversion with and without crossing-over; (GC)), BIR and chromosome loss (Loss) following DSB induction in diploid strains with and without IRs. Results of 3 to 10 experiments were used to calculate the median frequencies and the ranges of the medians [in brackets] of GC, BIR and Loss outcomes. Asterisks indicate statistically significant (P <0.01) changes compared to the No-IR strain.

To test this idea, several diploid strains were generated by crossing *MAT****a*** strains carrying IRs (IR-12 or IR-1000) or not carrying IRs, with a *MATα-inc* strain (AM476, S1 Table), which contains a mutation (*α-inc)* that prevents from cutting of *MAT* by HO endonuclease (similar to [48], Fig 7A-i). In addition, the *MATα-inc* strain did not contain IRs, but carried a *URA3* gene inserted at the position of the *THR4* gene. DSBs were initiated at *MAT***a** by plating cells on a galactose-containing media, and the repair outcomes were analyzed (see Materials and Methods for details) and classified (Fig 7A- ii, iii, iv). We observed that in the absence of IRs, DSB repair predominantly led to GC (>90%) with *MAT***a** replaced by *MATα-inc*. Of all repair events, approximately 5% of repair events had phenotypes indicative of BIR. In particular, they were represented by either Ade^+^ Thr^-^ Ura^+^ full colonies or by sectored colonies, where one sector was Ade^+^ Thr^-^ Ura^+^ while another was Ade^+^ Thr^+^ Ura^+^ (Fig 7A-iii and 7B-BIR). In addition, <0.5% of all events were Ade^-^Thr^-^Ura^+^ and resulted from the loss of the broken chromosome (Fig 7A-iv and 7B-Loss).

**Fig 8:**
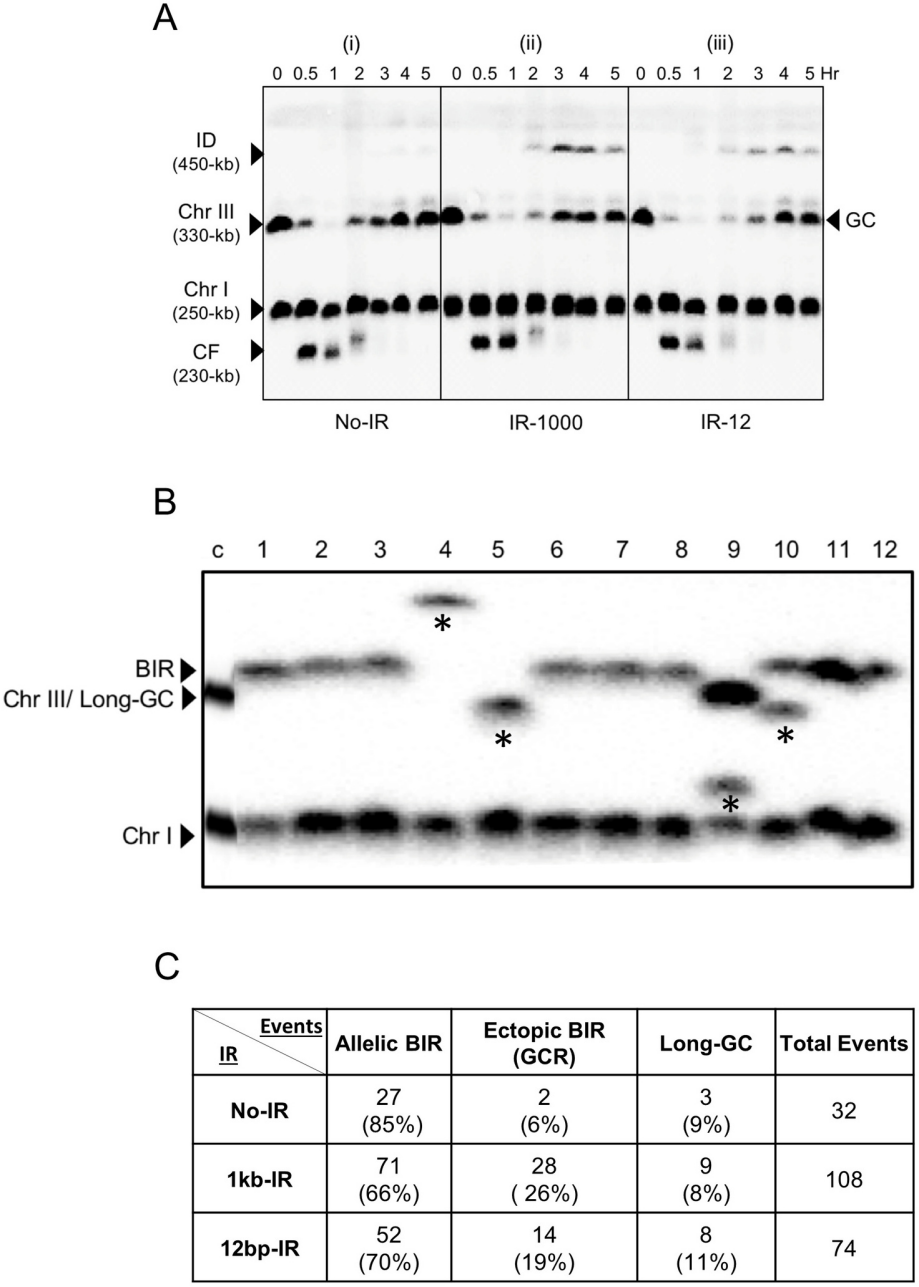
The effect of IRs on DSB repair in diploids. **(A)** Kinetics of DSB repair in diploid strains with IR-1000, IR-12 or No-IR analyzed by CHEF gel electrophoresis followed by Southern hybridization using *ADE1*-specific probe (hybridized to Chr III and Chr I). A repair product of ~450-kb size (inverted dimer) was detected starting from 2 hours following DSB induction in IR-1000 and IR-12 diploid strains. The fragment labeled CF is the Chr III cut-fragment resulting from HO-induced DSB. An additional (higher) CF band, observed 2-hr after the addition of galactose, corresponds to the processed (partially single-stranded) form of the cut fragment (similar to [86]). **(B)** The structure of Chr III in BIR outcomes (Ade^+^ Thr^-^ Ura^+^) obtained from diploids containing IR-12 by CHEF gel electrophoresis, followed by Southern analysis using *ADE1*-specific probe. First lane (c): control of DNA before DSB induction. Few examples of outcomes containing GCR (resulting from ectopic BIR) are indicated by asterisks. **(C)** The number of allelic BIR, ectopic BIR, and long gene conversion (Long-GC) events among representative Ade^+^ Thr^-^ Ura^+^ outcomes as distinguished by CHEF electrophoresis followed by Southern hybridization using *ADE1*-specific probe.

We observed a 3-fold increase of BIR events in diploids containing inverted repeats (IR-1000 or IR-12) in the vicinity of a DSB as compared to the control without inverted repeats (Fig 7B-BIR). The presence of IRs also led to a significant increase of chromosome loss (approximately 4% of chromosome loss in IR-1000 and IR-12 versus no detected cases of chromosome loss in no-IR strain (Fig 7B-Loss)), which suggested that the repair of DSBs introduced in the vicinity of IRs was more likely to fail as compared to strains without IRs. Overall, our results suggested that IRs located in the vicinity of a break channel some DSB repair from GC into BIR and increased chromosome loss. We hypothesized that the decrease of GC resulted from IR-mediated SSA disrupting the coordinated behavior of two broken DSB ends, which is known to be important for the successful GC repair [26]. This hypothesis was supported by our observation of a concomitant accumulation of IDs along with GC repair products that we observed in a time-course experiment performed in IR-1000 diploid strain (Fig 8A). This observation confirmed that inter-molecular SSA can indeed successfully compete with allelic GC repair. Interestingly, ID formation was observed in the IR-12 diploid as well, where they could be either formed by inter-molecular SSA or by replication of unprocessed FB structures. The latter possibility is less likely since no accumulation of FBs was observed in this strain, which suggested that FBs were efficiently processed by the MRX-Sae2 complex.

We previously demonstrated that inverted dicentric dimers resulting from inter-molecular SSA between IRs can break in mitosis and the broken fragments are often repaired by ectopic BIR using non-homologous chromosomes generating translocations ([17, 49]). Here we asked whether the BIR occurring in diploids containing IR-1000 or IR-12 was allelic or ectopic. Allelic BIR proceeds via invasion of a broken fragment into the homologous chromosome at an allelic position. Ectopic BIR proceeds by strand invasion into a non-homologous chromosome at repeated sequences such as Ty or delta elements. CHEF-gel electrophoresis analysis of DNA isolated from Ade^+^ Thr^-^ Ura^+^ repair outcomes (Fig 7B-BIR) from IR-containing diploids revealed that 66% (71/108) and 70% (52/74) resulted from allelic BIR in IR-1000 and IR-12 strains respectively (Fig 8B and 8C). Further, 26% (28/108) and 19% (14/74) of analyzed repair outcomes in IR-1000 and IR-12, respectively, contained an unusually sized *ADE1*- hybridizing band indicative of ectopic BIR repair (similar to [13, 17]) (Fig 8B and 8C). The frequency of ectopic BIR was slightly less (6%) in No-IR strain as compared to strains with IR, even though the increase of ectopic BIR proved to be statistically significant only in a case of IR-1000 as compared to No-IR strain (P = 0.02 by Fisher’s exact test). Surprisingly, our CHEF analysis of Ade^+^ Thr^-^ Ura^+^ events also demonstrated that approximately 10% of these outcomes contained repair band that was similar in size to the original, recipient chromosome (Fig 8B and 8C-Long-GC), and therefore could represent cases with a very long (>16-kb) gene conversion, known to be mechanistically similar to BIR [19].

## Discussion

Inverted DNA repeats are a known source of chromosomal rearrangements including those leading to cancer (reviewed in [50, 51]), Emanuel syndrome, and X-linked congenital hypertrichosis. Two molecular mechanisms promoting IR-mediated genomic destabilization include DNA breaks introduced into secondary DNA structures formed by IRs as well as the interruption of DNA replication at the positions of secondary structures formed by IRs, both of which have been the subject of multiple studies [15, 16, 18, 28, 52]. In the present study, we provide several insights into, yet another mechanism of genetic destabilization mediated by IRs, where IRs mis-route the repair of DSBs introduced in their vicinity. First, we demonstrate that such DSBs can be channeled into two types of IR-mediated single-strand annealing: intra-molecular SSA and inter-molecular SSA and that the choice between these two pathways is dictated by the size of the DNA spacer separating two halves of the IR. Second, we demonstrate that different protein complexes participate in the processing of FB structures and the choice of the protein complex is dictated by the distance between the two arms of the IRs. Finally, we demonstrate that both IR-mediated mechanisms channel DSB repair into pathways that are prone to genomic rearrangements.

### Genomic rearrangements initiated by IR-mediated mis-routing of DSB repair

**Fig 9:**
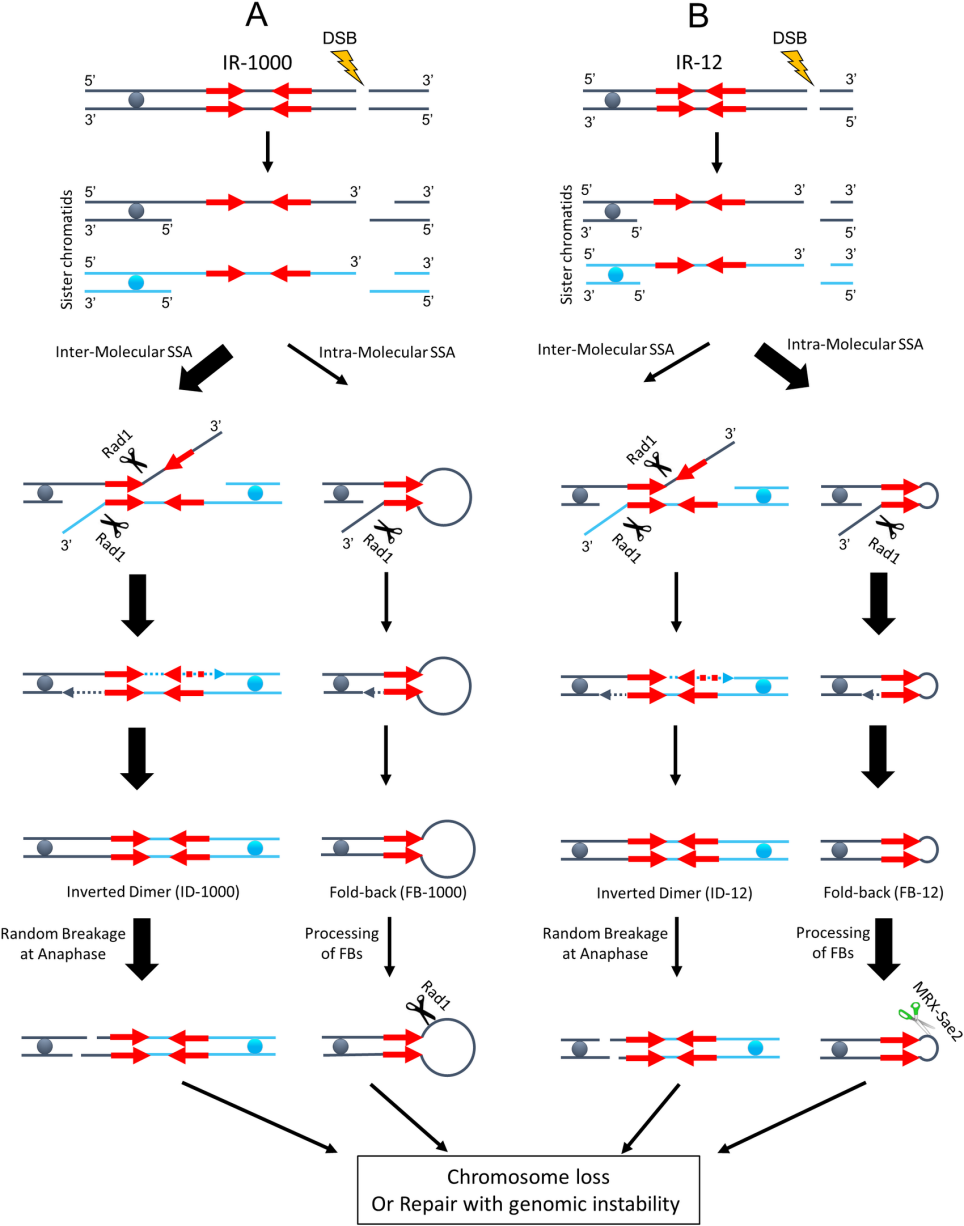
DSB-induced SSA involving inverted repeats. **(A)** In strains with IRs separated by long (1-kb) spacers (IR-1000), inter-molecular SSA (involving IRs in different sister chromatids) is the predominant SSA mechanism and led to the formation of inverted dicentric dimers (ID-1000). **(B)** In a case of IR separated by small (~12-bps) spacers (IR-12), intra-molecular SSA leading to the formation of fold-backs (FBs) predominates. The preferred pathway in each case is indicated by a thicker black arrow. Rad1 is involved in clipping of 3’-flaps during the ID and FB formation. Breakage of dicentrics in mitotic anaphase leads to the repair by BIR or chromosome-loss leading to genomic instability. FB-12 are processed by MRX-Sae2 complex, while FB-1000 are processed by Rad1. Processing of FBs can also lead to GC, BIR or chromosome loss and eventually to genomic instability.

According to our model (Fig 9A and 9B), 5’- to 3’ resection of DSB ends allows inter-molecular or intra-molecular SSA between IRs leading to the formation of inverted dicentric dimers or fold-back structures. The 3’ clipping activity of Rad1-Rad10 complex is required during both of these processes, but these two pathways differ from each other in their level of Rad1-Rad10 dependency. In particular, the inter-molecular SSA requires clipping of two non-homologous 3’-flaps (Fig 9A and 9B; Inter-molecular SSA pathway), which cannot proceed without Rad1-Rad10, thus making inter-molecular SSA fully Rad1-Rad10 dependent. However, formation of fold-backs that requires cleavage of only one 3’-flap, shows only partial dependence on Rad1-Rad10 complex. This observation indicated that besides Rad1-Rad10 complex, some other protein (s) can perform 3’ flap cleavage, which is consistent with several other studies [53, 54]. At present, the identity of this hypothetical protein remains unknown. Based on our results, it seems unlikely that this protein is Rad2, Slx1, Yen1, or Mus81 though we cannot exclude that several nucleases from this list may perform this function in a redundant fashion.

The predominance of intra-molecular SSA for IRs separated by short (12-bp) rather than long (1-kb) spacer might be explained by a higher probability of interaction for IRs in close proximity to each other as compared to those located further away. Another possible contributing factor is thermodynamic stability of FB structures, which was shown to be inversely proportional to the length of the spacer separating IRs [55, 56]. Importantly, this data is consistent with the results obtained by [57–59] who previously demonstrated that the efficiency of intra-molecular annealing between IRs is inversely proportional to the distance between them. The facilitation of inter-molecular SSA between IRs separated by longer spacers may be promoted by sister chromatid cohesion, which is known to facilitate inter sister recombination [60, 61], while shorter spacers could make it difficult for IRs located on different sisters to contact each other. Importantly, our data demonstrates that Rad52 is involved in the formation of FBs containing both long and short spacers, which refuted a previously formulated hypothesis that the formation of FBs separated by short spacers could be Rad52-independent [16]. The Rad52 dependence (and Rad51-independence) of FB formation, is consistent with an SSA pathway responsible for FB formation requiring a direct involvement of Rad52 in the annealing between ssDNA regions.

We propose that when FBs are formed, they can be processed by two different protein complexes. Specifically, FBs containing short single-strand loops are processed by MRX-Sae2 complex (consistent with previous observations [16, 62, 63]), while FBs with long spacers (≥ 1-kb) are processed by Rad1-Rad10 complex. Interestingly, two nucleases appear partially redundant when it comes to processing of FBs with intermediate-length spacers. We speculate that longer ssDNA spacers appear to be similar to a long 3’-flap that therefore can be recognized and cut by the Rad1-Rad10 complex. Previously, it has been shown that binding of RPA to ssDNA stimulates the cutting activity of XPF protein, a human homolog of Rad1 [64, 65]. Perhaps the binding of larger ssDNA loops by RPA can stimulate the activity of Rad1-Rad10 complex. Conversely, RPA binding might inhibit processing of ssDNA by MRX-Sae2 protein complex. Together, RPA binding to the larger ssDNA loops might play a role in the choice of proteins for ssDNA loop processing. However, it remains unclear how different loops of larger size are distinguished from each other. For example, both 500-bp and 1000-bp loop are expected to be bound by RPA; yet the former can be cut by MRX-Sae2 complex, while the latter not, thus suggesting the existence of some other, presently unknown, factors contributing to the distinction between these loops in respect to their recognition by MRX-Sae2 complex, even though both can be processed by Rad1-Rad10 complex. Finally, our data obtained in diploid cells suggests that both types of IR-mediated SSA (inter- and intra-molecular) cause shifting of DSB repair from GC into BIR. To explain this, we propose that a DSB end proximal to IRs is frequently engaged in IR-mediated SSA which disrupts coordinated action of the two broken DNA ends required for successful GC. In particular, we propose that inter-molecular SSA leads to the formation of inverted dicentric dimers, which are subsequently broken during mitotic division (Fig 9). The new broken ends are essentially “one-ended” and therefore prone to repair via BIR (reviewed in [8]). In a case of FB formation, the increased BIR can be explained by one of two possible scenarios. The FB could be processed by either MRX-Sae2 or by Rad1-Rad10 nuclease complexes, and the resulting broken end can be repaired by either GC (if the second end of the original, HO-induced DSB end is still available), or by BIR (if the second DSB end is degraded or engaged in another recombination event). Alternatively, if the FB remains unprocessed, then following DNA replication, it could be converted into an ID (similar to [18, 28]). The latter possibility is consistent with the results of our time-course analysis using diploid strains containing IR-12, where significant accumulation of IDs was detected.

### The effect of inverted repeats on DSB repair: Implications for understanding genomic instabilities in humans

Here we characterized two pathways of DSB repair involving annealing between IRs that can result in genomic destabilization. IRs are especially frequent in the 17% of the human genome comprised of LINEs and Alu repeat elements that commonly form clusters, where individual repeats often have opposite orientations [66, 67]. Alu elements (~300-bp in size) are the most abundant class of large dispersed DNA repeats in humans, while LINEs (~6-kb in size) are autonomous transposable elements that are present in thousands of copies in human genome [68, 69]. LINEs correlate in size of repeat to yeast Ty elements (~6-kb) that are responsible for the majority of IR-mediated GCRs reported in yeast [17, 18, 32, 34, 57]. Additionally, Alu elements promote genome destabilization similar to that induced by yeast Ty elements [58]. The length of IRs used in our study was within the length of naturally occurring IRs as well as within the lengths used previously by various groups to study IR-mediated GCRs, as well as to investigate the genetics of SSA [17, 18, 24, 25, 32, 36, 37, 57–59]. In addition, the majority of previous studies believed that only IR pairs separated by very short (<20-bp) spacers promote high levels of genomic instabilities [58, 68, 70]. However, clusters of repeat elements do not always have spacers of the short <20-bp size [66, 67]. The results obtained in our study also point towards genome destabilization that can result from IRs separated by much longer (~ 1kb) spacers.

With IRs being abundant in the human genome (see for example in [28, 50, 71]), we propose that the mechanisms described here are likely to occur in human cells. Specifically, it has been demonstrated that DNA amplifications described in various human cancers often are associated with IRs [50, 72–74]. Further, genetic destabilizations observed in cancer cells often result from mis-routed DSB repair (reviewed in [75, 76]), so it is possible that gene amplifications and/or genomic destabilizations observed in cancer cells can result from inter- or intra-molecular SSA between IRs. Additionally, it is possible that inter- or intra-molecular SSA between IRs induced by DSBs can contribute to genetic rearrangements leading to several other human diseases. For example, Emanuel syndrome is known to result from recurrent and non-recurrent translocations induced by palindromic AT-rich repeats (PATRRs) [77]. These translocations result from DNA breaks introduced in the middle of the palindrome, likely at the tip of a secondary structure (cruciform or hairpin formed by the palindrome) (reviewed in [78]). We propose that the mechanism described in our study can facilitate formation of secondary structures by PATRR, which in turn can facilitate breakage leading to formation of translocations. It is also possible that the molecular mechanisms described here could contribute to the formation of β-thalassemia, known to originate from large palindrome-induced deletions [30].

In the future, it will be important to compare the structural properties of inverted DNA repeats promoting different disease-associated rearrangements, since it may reveal that different IR-mediated SSA pathways are involved in their formation. It is also important to characterize the respective roles of human homologs of Rad1-Rad10 and MRX-Sae2 complexes in the formation of DSB and IR-promoted chromosomal rearrangements since it may shed light on the specific mechanisms of chromosomal rearrangements leading to various pathologies in humans.

## Materials and methods

### Yeast strains

The genotypes of all strains used in this study are shown in S1 Table. The haploid yeast strain AM1102 contains two 2-kb long *PHO87* sequences in inverted orientation to each other, separated by a 1-kb-long spacer, and located 3-kb centromere proximal to *MAT***a**. AM1102 was derived from JKM111 (S1 Table) in three steps using *delitto perfetto* approach [79]. First, AM1036 was constructed by transformation of JKM111 with a DNA fragment generated by PCR amplification of the pGSKU plasmid [79] using two primers:

OL556: 5’AGATTGGGAGTTGGTAGACCTTTTGGTCGTTAATGAAATTGAGGGTCTTCtagggataacagggtaatccgcgcgttggccgattcat-3’

and OL557: 5’-GTACTTCAGGGCTTTCGTGCGAACAGAAAAGCACCCCTCTCGAACCCAAAttcgtacgctgcaggtcgac-3’

Upper-case letters correspond to the sequences upstream of the position 193891-bp and downstream of 194040-bp of chromosome III (according to *Saccharomyces cerevisiae* genome database). Lower-case letters correspond to the sequences specific to the pGSKU cassette [79]. Subsequently, AM1050 was constructed by transforming AM1036 with a DNA fragment generated by PCR amplification of the *PHO87* region using genomic DNA of JKM111 as a template. The primers used for the amplification were as follows:

OL554: 5’-AGATTGGGAGTTGGTAGACCTTTTGGTCGTTAATGAAATTGAGGGTCTTCctcacactttctcaaatacaacgc-3’;

and OL555: 5’-GTACTTCAGGGCTTTCGTGCGAACAGAAAAGCACCCCTCTCGAACCCAAAccagccgattccataaggttttaa-3’

Upper-case letters correspond to the sequences upstream of 193891-bp and downstream of 194040-bp positions of chromosome III, respectively. The lower-case letters correspond to the sequences specific to the *PHO87* region. This transformation resulted in formation of a 2-kb-long inverted repeat with a 1-kb-long spacer sequence separating IRs located 3-kb centromere proximal from *MAT***a**.

Subsequently, a series of strains containing the same 2-kb long repeat of *PHO87*, but separated by shorter spacers of various lengths were constructed by *delitto perfetto* protocol in two steps [79]. First, AM1354 was constructed by transformation of AM1050 with a DNA fragment generated by PCR amplification of the plasmid pGSKU using primers:

OL1107: 5’-TTCATTGACCATTCAAAGAAAAGGTGCTGCTGAAAGCATGCCACTGTATAAAGATGTTCAGAtagggataacagggtaatccgcgcgttggccgattcat-3’

and OL1108: 5’-GGTGTGTAGAGTCACAAATAGAAAGTGCTTTTGGATCGTCCGGTGAAATTGCAGTAATACttcgtacgctgcaggtcgac-3’.

The upper-case letters correspond to the sequences located upstream of 194287-bp and downstream of 194950-bp position of chromosome III and located inside the spacer separating inverted repeats in AM1050. Lower case letters correspond to the sequences specific to the pGSKU cassette.

Subsequently, a series of strains with 500-bp (AM1398), 100-bp (AM1399) and 12-bp (AM1648) spacer separating IRs were constructed by deleting the pGSKU cassette and varying amounts of spacer DNA sequence. This was carried out by transformation of AM1354 with following denatured oligonucleotides as described in [79]:

For the construction of AM1398 with 500-bp-long spacer between 2-kb inverted repeats:

OL1258: 5’-GTATTGCACATGAACACTAGGCAAGAATTAATAGAAAGTGAATGGAATGGCTTCACTAGCCCATAAGAAAGCACAGCATTCTAC-3’

For construction of AM1399 with 100-bp-long spacer between 2-kb inverted repeats:

OL1135: 5’-CGAGAGGGGTGCTTTTCTGTTCGCACGAAAGCCCTGAAGTACCACAGCATCGATAGAATACCTGTAGGCAGAGCGACAGCAAAA-3’

For construction of AM1648 with 12-bp-long spacer between 2-kb inverted repeats:

OL1214: 5’-TCCATGCAGTACTTAAAACCTTATGGAATCGGCTGGTTTGGGTTGCCCCCAGCCGATTCCATAAGGTTTTAAGTACTGCATGGA-3’

Finally, AM1757, AM1760 & AM1762 were constructed from AM1398, AM1399 and AM1648 respectively by transformation with *ade3*::*GAL*::*HO* cassette using pop-in-pop-out approach [80].

AM2290 and AM2295 were constructed from AM1102 using *delitto perfetto* approach in two steps. First, AM1352 was constructed from AM1102 by inserting pCORE cassette into the spacer region between 2-kb IRs. This was carried out by transforming AM1102 with DNA fragment generated by PCR amplification of the spacer DNA sequence and pCORE plasmid [79] using the following primers:

OL1109: 5’–TTCATTGACCATTCAAAGAAAAGGTGCTGCTGAAAGCATGCCACTGTATAAAGATGTTCAGAgagctcgttttcgacactgg– 3’

OL1110: 5’–GTGTGTAGAGTCACAAATAGAAAGTGCTTTTGGATCGTCCGGTGAAATTGCAGTAATACtccttaccattaagttgatc– 3’

Upper-case letters correspond to the sequences upstream of 194287-bp position and downstream of 194950-bp position on chromosome III. The lower-case letters correspond to the sequences specific to the pCORE cassette. Finally, AM2290 and AM2295, with inverted repeats separated by bacteriophage lambda spacers that were 1-kb (in AM2290) or 1.5-kb (in AM2295) in length were constructed by transformation of AM1352 with DNA fragments generated by digestion of plasmids–pAM-36 & pAM-37 with HindIII and EcoRI restriction enzymes, generating fragments containing 1-kb or 1.5-kb DNA sequence from bacteriophage lambda DNA in between *PHO87* inverted repeat sequence.

The majority of single-gene deletion mutants were constructed by transformation with a PCR-derived *KAN-MX* module (see the strain list in S1 Table for genes deleted by *KAN-MX*) flanked by terminal sequences homologous to the sequences flanking the open reading frame of each gene [81]. The resulting constructs were confirmed by PCR and by phenotype analysis. To disrupt *RAD1*, a pJH551plasmid was digested with SalI and transformed into recipient strains, and transformants were selected on a leucine-dropout media. To construct AM1362, AM1401, AM2207, *RAD52* was disrupted using plasmid pJH181 that was digested with BamHI and transformed into recipient strains, and transformants were selected on a leucine-dropout media. The full list of oligonucleotides used in this study is available upon request.

### Growth conditions

Yeast were grown at 30°C. Yeast extract-peptone-dextrose (YEPD) media, and synthetic complete medium, with nitrogenous bases or amino acids omitted were prepared as described in [82]. YEP-lactate (YEP-lac) and YEP-galactose (YEP-gal) contained 1% yeast extract and 2% Bacto peptone that was supplemented with either 3.7% lactic acid (pH 5.5) or 2% (w/v) galactose, respectively. 5-fluoroorotic acid (5-FOA) was added to synthetic complete medium with trace amounts of uracil (30mg/mL). For selection of cells containing insertion of *KAN*::*MX* cassette G418 (Calbiochem) was added to YEPD media to the final concentration of 0.3 g/L. The arrest of cells at G1 cell cycle stage was achieved by addition of α-factor (Zymo research) to the final concentration of 5μM.

### Distribution of DSB repair events

To determine the distribution of DSB repair events in yeast diploids, yeast cultures were grown in leucine drop-out medium for ~20 hours, followed by ~18 hours in YEP-lactate, and then diluted and plated on YEP-Gal plates similar to [13, 48]. Colonies that grew on YEP-Gal plates were then replica plated onto appropriate omission media to determine the fraction of DSB repair events with the following phenotypes: (i) Ade^+^ Thr^+^ Ura^+^ (gene conversion; GC) (ii) Ade^+^ Thr^+/-^ Ura^-/+^ (GC associated with crossing-over); (iii) Ade^+^ Thr^-^ Ura^+^ (BIR); (iv) Ade^-^ Thr^-^ Ura^+^ (chromosome loss). The frequency of individual DSB repair outcomes were determined as described in [48] based on at least three experiments. Cell viability following HO induction was derived by dividing the number of colony-forming units (CFUs) on YEP-Gal by the number of CFUs on YEPD.

### Analysis of DSB repair kinetics

The kinetics of DSB repair was analyzed in experiments where cell culture aliquots were removed every 30 or 60 minutes following galactose addition, similarly to described previously [20, 48, 83]. In brief, yeast cells were grown for approximately 20 hours in YEP-lac media to a final concentration of approximately 1–2 x 10^7^ cells per ml. To induce HO breaks, galactose was added to the final concentration of 2%. We used 50-ml culture aliquots for the extraction of DNA by glass-bead-phenol-sodium dodecyl sulfate protocol [84] that was used for Southern blot analysis. For CHEF gel electrophoresis, chromosomal plugs were prepared as described in [48]. For Southern blot analysis, the DNA was digested with appropriate restriction enzymes, and the resulting fragments were separated using 1% agarose gel. CHEF was performed using genomic DNA embedded in plugs of 1% agarose. The DNA was subsequently analyzed using Southern blot analysis. The membranes containing separated DNA fragments were hybridized to the chromosome-specific DNA probes, produced by labeling of chromosome-specific DNA fragments with P^32^. The *ADE1*-specific probe was prepared using pJH879 plasmid digested with SalI [85]. All other probes were generated by PCR amplification using genomic DNA of AM1102 as a template and were specific to the following yeast sequences: 1) Probe P-1 specific to *RBK1* region of chromosome III located between 193591–193891 bps positions. 2) Probe P-2, specific to PHO87-IR specific located between 196893–197172 bps positions of chromosome III. 3) Probe P-3, specific to *BUD5* region of chromosome III located between 197181–197488 bps positions; 4) Probe P-4, specific to the *IRC7* region of chromosome VI located between 265004–265341 bps positions. The chromosomal coordinates for these probes were derived from SGD. The full list of oligonucleotides that were used to generate each probe is available upon request. Blots were analyzed by using ImageQuant TL 7.0 software from GE healthcare. The amounts of DSB repair products obtained by combination of CHEF and Southern blot analysis were calculated as the percentage of original chromosome intensity before DSB induction that was converted to the repair product. Quantification of the fold-back (FB) and Inverted Dimer (ID) levels in the restriction digestion analysis were calculated as the percentage of cut fragment-CF (detected at 0.5-hr time point) that was converted into FBs and IDs respectively. All the gels were run with appropriate molecular-size markers that allowed to estimate the size of every band visualized in every image. All quantifications were based on the results of at least three independent experiments.

## Supporting information

S1 Table Strain List. The list of yeast strains used in this study.
(PPTX)

S1 Fig **(A)** Efficiency of ID formation in various strain backgrounds. Efficiency of ID formation (%) calculated by dividing the normalized intensity of ID band present at 6 hours following DSB induction by the intensity of cut-fragment generated at 0.5-hr following DSB in IR-1000, IR-500, IR-100, and IR-12 strains. The median of ID efficiency and the range [in the brackets] calculated based on a minimum or 3 experiments are indicated above each strain. **(B)** The schematics of AvrII digest of Chr III (OF) in IR-1000 and its DSB-derivatives, CF, ID, FB. The location of probe P-3 (specific to *BUD5* sequence) is indicated by green box. **(C)** Southern blot analysis of DSB repair in IR-1000-*rad1Δ* following AvrII digestion and hybridization with probe P-3. **(D)** Efficiency of FB formation (%) calculated by dividing the normalized intensity of FB band formed at 6-hrs following DSB induction by the intensity of cut-fragment generated at 0.5-hr following DSB in various strains. The median of FB formation and the range [in the brackets] calculated based on minimum of 3 experiments are indicated above each strain. The cut-fragment band (0.5-hr time-point post DSB induction) was used for calculation because it represented the actual amount of chromosomes that were broken following DSB induction and therefore could be involved in DSB repair. In addition, the Southern transfer of the original (uncut) chromosome fragment (OF) at 0-hr time-point (before DSB) was inefficient and therefore was unreliable for calculations.
(TIF)

S2 Fig (**A)** The schematic of AvrII digest of Chr III (OF) in IR- λ1000 strain (two 2-kb IRs separated by 1000-bp spacer DNA derived from λphage-DNA) and its DSB-derivatives including: CF, ID, and FB. **(B)** Southern blot analysis of DSB repair in *RAD1* and *rad1Δ* derivatives of IR- λ1000 strain following hybridization to probe P-1. **(C)** The schematic of AvrII digest of Chr III (OF) in IR- λ1500 strain (two 2-kb IR separated by 1500-bp spacer DNA derived from λphage-DNA) and its DSB-derivatives including: CF, ID, and FB. **(D)** Southern blot analysis of DSB repair in *RAD1* and *rad1Δ* derivatives of IR- λ1500 strain following hybridization to probe P-1.
(TIF)
