# Supplementary material for: Single-strand annealing between inverted DNA repeats: Pathway choice, participating proteins, and genome destabilizing consequences
Source: PLoS Genet. 2018 Aug 9;14(8):e1007543. doi: 10.1371/journal.pgen.1007543 (PMC6103520; doi:10.1371/journal.pgen.1007543)
Supplement: S1 Table — The list of yeast strains used in this study. (PPTX) [file pgen.1007543.s001.pptx]

## Slide 1
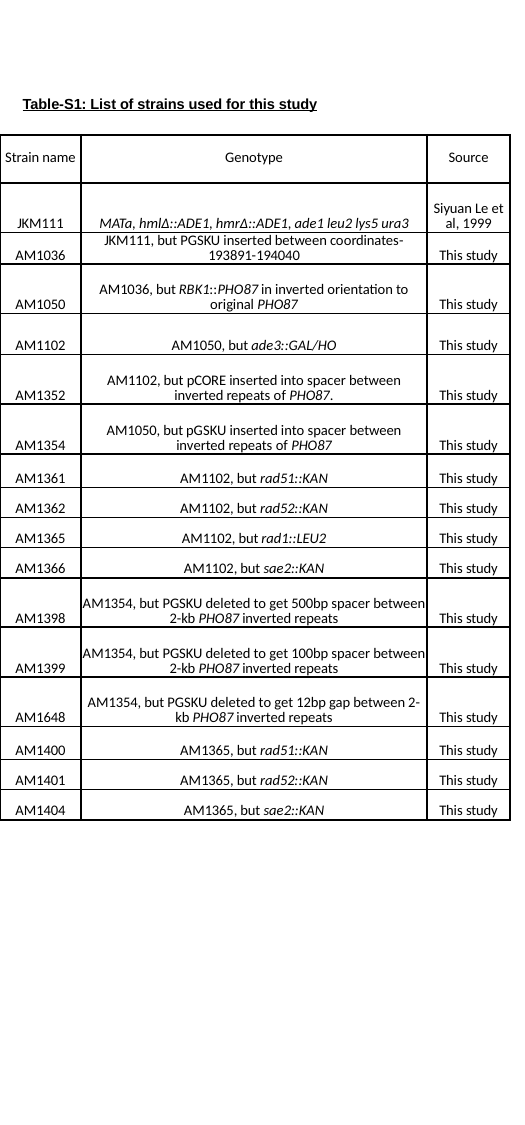

Table-S1: List of strains used for this study
| Strain name | Genotype | Source |
| --- | --- | --- |
| JKM111 | MATa, hml∆::ADE1, hmr∆::ADE1, ade1 leu2 lys5 ura3 | Siyuan Le et al, 1999 |
| AM1036 | JKM111, but PGSKU inserted between coordinates-193891-194040 | This study |
| AM1050 | AM1036, but RBK1::PHO87 in inverted orientation to original PHO87 | This study |
| AM1102 | AM1050, but ade3::GAL/HO | This study |
| AM1352 | AM1102, but pCORE inserted into spacer between inverted repeats of PHO87. | This study |
| AM1354 | AM1050, but pGSKU inserted into spacer between inverted repeats of PHO87 | This study |
| AM1361 | AM1102, but rad51::KAN | This study |
| AM1362 | AM1102, but rad52::KAN | This study |
| AM1365 | AM1102, but rad1::LEU2 | This study |
| AM1366 | AM1102, but sae2::KAN | This study |
| AM1398 | AM1354, but PGSKU deleted to get 500bp spacer between 2-kb PHO87 inverted repeats | This study |
| AM1399 | AM1354, but PGSKU deleted to get 100bp spacer between 2-kb PHO87 inverted repeats | This study |
| AM1648 | AM1354, but PGSKU deleted to get 12bp gap between 2-kb PHO87 inverted repeats | This study |
| AM1400 | AM1365, but rad51::KAN | This study |
| AM1401 | AM1365, but rad52::KAN | This study |
| AM1404 | AM1365, but sae2::KAN | This study |

## Slide 2
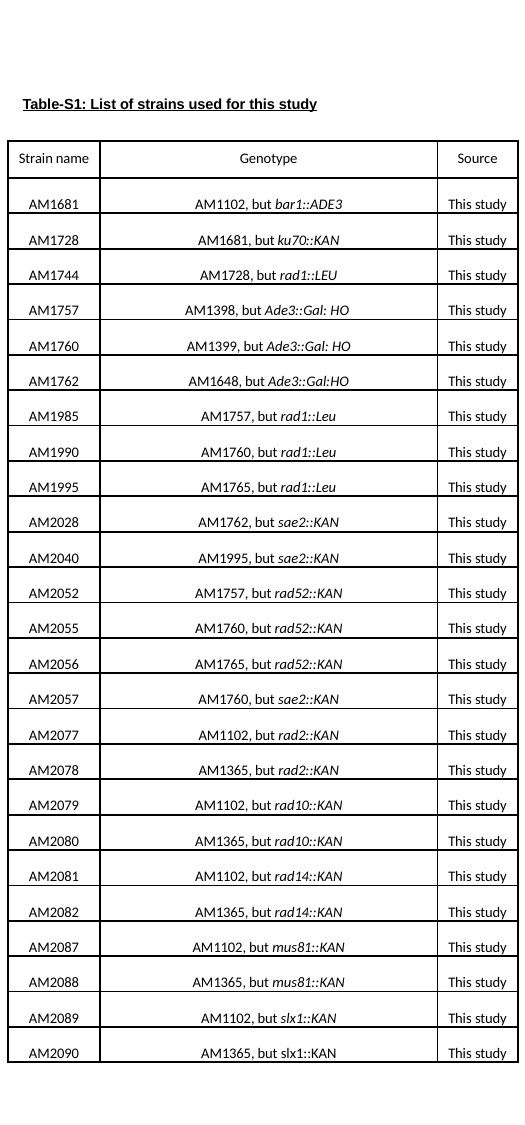

Table-S1: List of strains used for this study
| Strain name | Genotype | Source |
| --- | --- | --- |
| AM1681 | AM1102, but bar1::ADE3 | This study |
| AM1728 | AM1681, but ku70::KAN | This study |
| AM1744 | AM1728, but rad1::LEU | This study |
| AM1757 | AM1398, but Ade3::Gal: HO | This study |
| AM1760 | AM1399, but Ade3::Gal: HO | This study |
| AM1762 | AM1648, but Ade3::Gal:HO | This study |
| AM1985 | AM1757, but rad1::Leu | This study |
| AM1990 | AM1760, but rad1::Leu | This study |
| AM1995 | AM1765, but rad1::Leu | This study |
| AM2028 | AM1762, but sae2::KAN | This study |
| AM2040 | AM1995, but sae2::KAN | This study |
| AM2052 | AM1757, but rad52::KAN | This study |
| AM2055 | AM1760, but rad52::KAN | This study |
| AM2056 | AM1765, but rad52::KAN | This study |
| AM2057 | AM1760, but sae2::KAN | This study |
| AM2077 | AM1102, but rad2::KAN | This study |
| AM2078 | AM1365, but rad2::KAN | This study |
| AM2079 | AM1102, but rad10::KAN | This study |
| AM2080 | AM1365, but rad10::KAN | This study |
| AM2081 | AM1102, but rad14::KAN | This study |
| AM2082 | AM1365, but rad14::KAN | This study |
| AM2087 | AM1102, but mus81::KAN | This study |
| AM2088 | AM1365, but mus81::KAN | This study |
| AM2089 | AM1102, but slx1::KAN | This study |
| AM2090 | AM1365, but slx1::KAN | This study |

## Slide 3
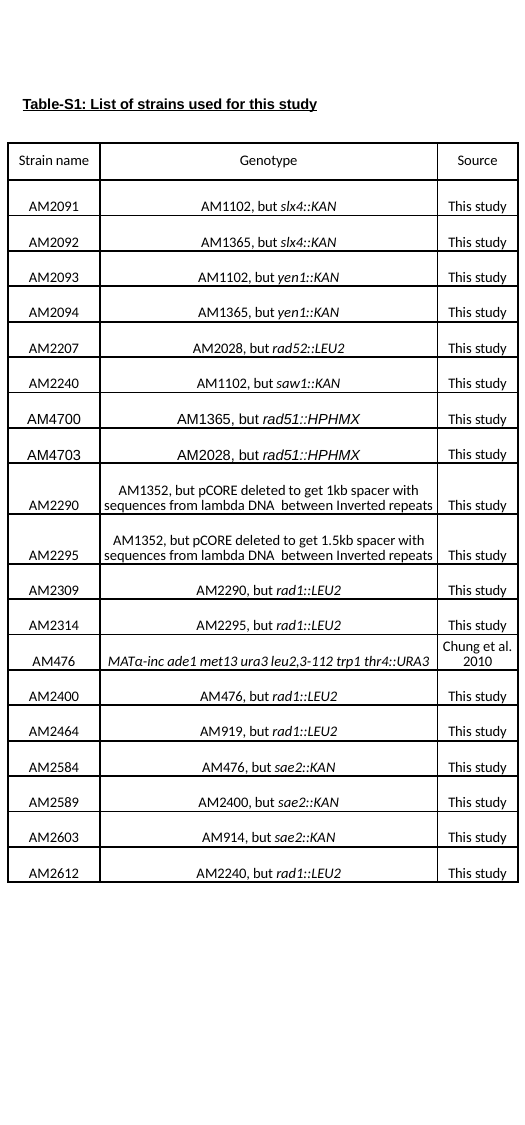

Table-S1: List of strains used for this study
| Strain name | Genotype | Source |
| --- | --- | --- |
| AM2091 | AM1102, but slx4::KAN | This study |
| AM2092 | AM1365, but slx4::KAN | This study |
| AM2093 | AM1102, but yen1::KAN | This study |
| AM2094 | AM1365, but yen1::KAN | This study |
| AM2207 | AM2028, but rad52::LEU2 | This study |
| AM2240 | AM1102, but saw1::KAN | This study |
| AM4700 | AM1365, but rad51::HPHMX | This study |
| AM4703 | AM2028, but rad51::HPHMX | This study |
| AM2290 | AM1352, but pCORE deleted to get 1kb spacer with sequences from lambda DNA between Inverted repeats | This study |
| AM2295 | AM1352, but pCORE deleted to get 1.5kb spacer with sequences from lambda DNA between Inverted repeats | This study |
| AM2309 | AM2290, but rad1::LEU2 | This study |
| AM2314 | AM2295, but rad1::LEU2 | This study |
| AM476 | MATα-inc ade1 met13 ura3 leu2,3-112 trp1 thr4::URA3 | Chung et al. 2010 |
| AM2400 | AM476, but rad1::LEU2 | This study |
| AM2464 | AM919, but rad1::LEU2 | This study |
| AM2584 | AM476, but sae2::KAN | This study |
| AM2589 | AM2400, but sae2::KAN | This study |
| AM2603 | AM914, but sae2::KAN | This study |
| AM2612 | AM2240, but rad1::LEU2 | This study |
