# Supplementary figures and images for: Single-strand annealing between inverted DNA repeats: Pathway choice, participating proteins, and genome destabilizing consequences
Source: PLoS Genet. 2018 Aug 9;14(8):e1007543. doi: 10.1371/journal.pgen.1007543 (PMC6103520; doi:10.1371/journal.pgen.1007543)

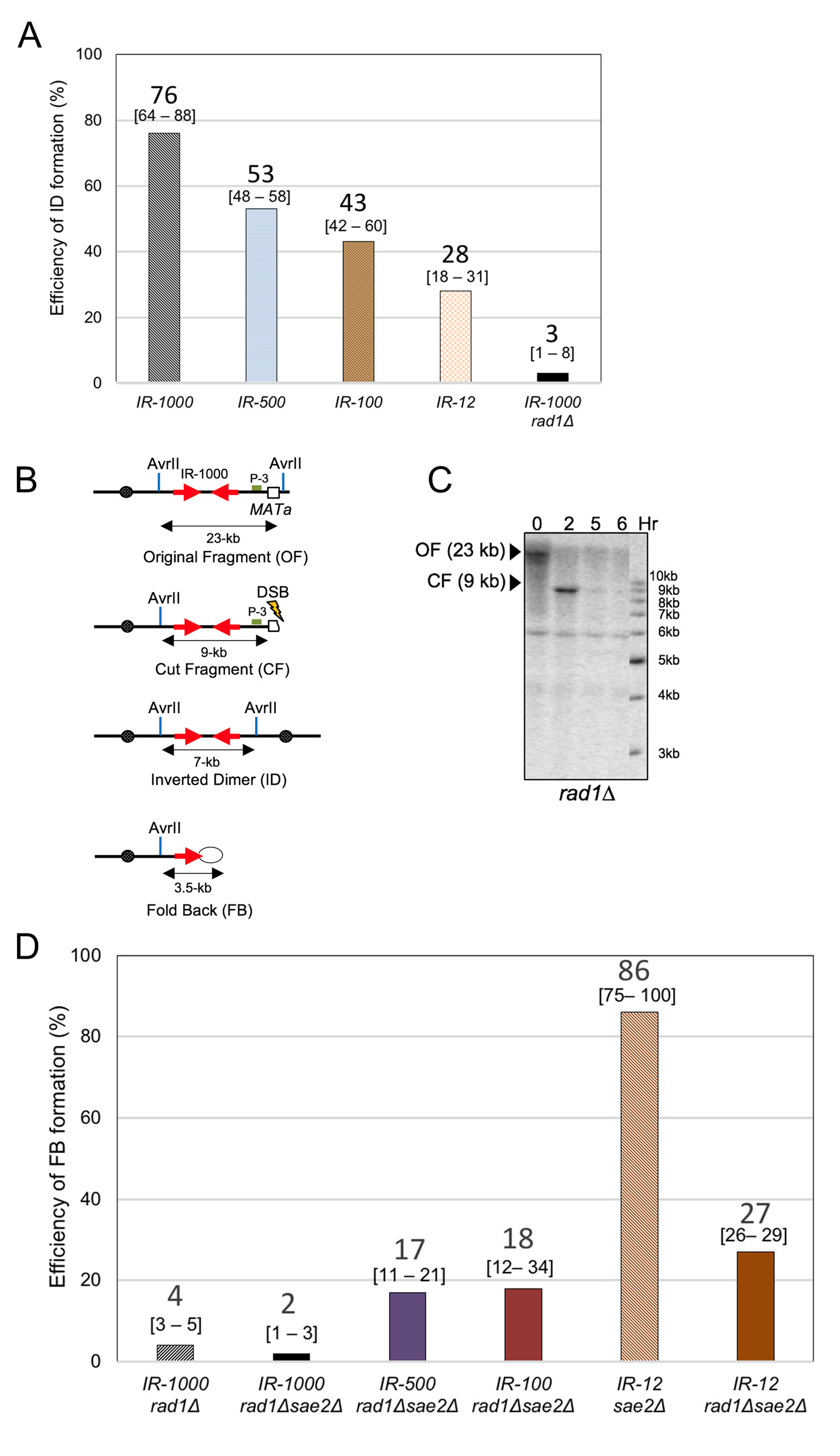

Supplement: S1 Fig — (A) Efficiency of ID formation in various strain backgrounds. Efficiency of ID formation (%) calculated by dividing the normalized intensity of ID band present at 6 hours following DSB induction by the intensity of cut-fragment generated at 0.5-hr following DSB in IR-1000, IR-500, IR-100, and IR-12 strains. The median of ID efficiency and the range [in the brackets] calculated based on a minimum or 3 experiments are indicated above each strain. (B) The schematics of AvrII digest of Chr III (OF) in IR-1000 and its DSB-derivatives, CF, ID, FB. The location of probe P-3 (specific to BUD5 sequence) is indicated by green box. (C) Southern blot analysis of DSB repair in IR-1000-rad1Δ following AvrII digestion and hybridization with probe P-3. (D) Efficiency of FB formation (%) calculated by dividing the normalized intensity of FB band formed at 6-hrs following DSB induction by the intensity of cut-fragment generated at 0.5-hr following DSB in various strains. The median of FB formation and the range [in the brackets] calculated based on minimum of 3 experiments are indicated above each strain. The cut-fragment band (0.5-hr time-point post DSB induction) was used for calculation because it represented the actual amount of chromosomes that were broken following DSB induction and therefore could be involved in DSB repair. In addition, the Southern transfer of the original (uncut) chromosome fragment (OF) at 0-hr time-point (before DSB) was inefficient and therefore was unreliable for calculations. (TIF) [file pgen.1007543.s002.tif]

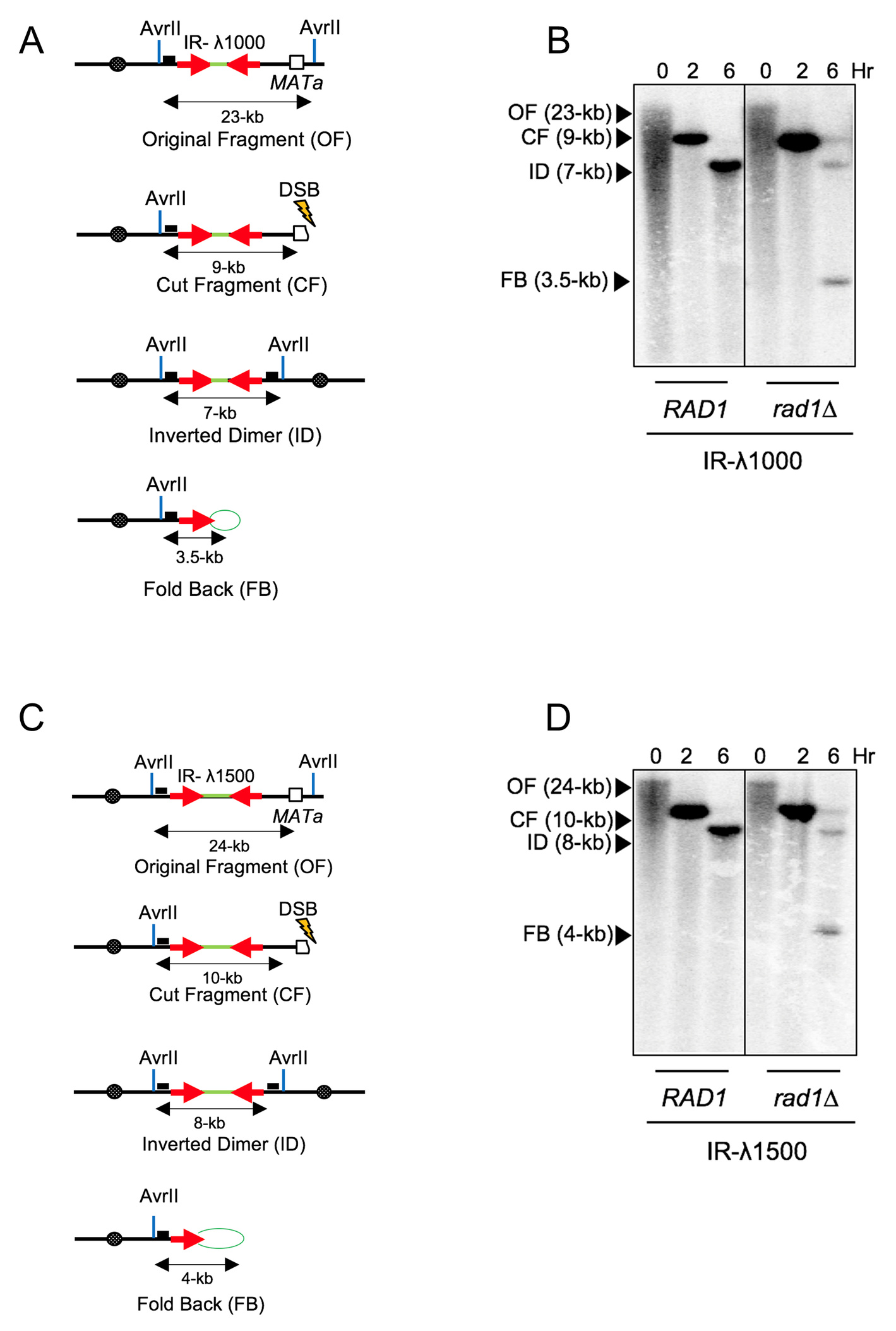

Supplement: S2 Fig — (A) The schematic of AvrII digest of Chr III (OF) in IR- λ1000 strain (two 2-kb IRs separated by 1000-bp spacer DNA derived from λphage-DNA) and its DSB-derivatives including: CF, ID, and FB. (B) Southern blot analysis of DSB repair in RAD1 and rad1Δ derivatives of IR- λ1000 strain following hybridization to probe P-1. (C) The schematic of AvrII digest of Chr III (OF) in IR- λ1500 strain (two 2-kb IR separated by 1500-bp spacer DNA derived from λphage-DNA) and its DSB-derivatives including: CF, ID, and FB. (D) Southern blot analysis of DSB repair in RAD1 and rad1Δ derivatives of IR- λ1500 strain following hybridization to probe P-1. (TIF) [file pgen.1007543.s003.tif]
